# Effects of alpha-glucosidase-inhibiting drugs on acute postprandial glucose and insulin responses: a systematic review and meta-analysis

**DOI:** 10.1038/s41387-021-00152-5

**Published:** 2021-03-03

**Authors:** Marjan Alssema, Carolien Ruijgrok, Ellen E. Blaak, Léonie Egli, Pierre Dussort, Sophie Vinoy, Jacqueline M. Dekker, M. Denise Robertson

**Affiliations:** 1 Former employee of Unilever Research and Development, Vlaardingen, The Netherlands; 2 Department of Epidemiology and Biostatistics, VU University Medical Center, Amsterdam Public Health Research Institute, Amsterdam, The Netherlands; 3 Department of Human Biology, NUTRIM, School of Nutrition and Translational Research in Metabolism, Maastricht University, Maastricht, The Netherlands; 4 Former employee of Nestle Research Center, Lausanne, Switzerland; 5 ILSI Europe, Brussels, Belgium; 6 Mondēlez International R&D, Nutrition Department, Saclay, France; 7 Department of Nutritional Sciences, University of Surrey, Guildford, UK

**Keywords:** Nutrition, Metabolism

## Abstract

**Background/objectives:**

Despite considerable literature supporting the potential health benefits of reducing postprandial glucose (PPG), and insulin (PPI) exposures, the size of a clinically relevant reduction is currently unknown. We performed a systematic review and meta-analysis to quantify effects of alpha-glucosidase-inhibiting (AGI) drugs on acute PPG and PPI responses.

**Methods:**

We searched EMBASE and MEDLINE until March 13, 2018 for controlled studies using AGI drugs together with a standardized carbohydrate load or mixed meal. The mean incremental PPG and PPI levels were calculated as outcomes. Meta-analyses, stratified by diabetes state, were performed by using random effects models.

**Results:**

The 66 included publications comprised 127 drug-control comparisons for PPG, and 106 for PPI, mostly testing acarbose or miglitol. The absolute effects on PPG were larger among individuals with diabetes (−1.5 mmol/l mean PPG [95% CI −1.9, −1.1] by acarbose, and −1.6 [−1.9, −1.4] by miglitol) as compared to individuals without diabetes (−0.4 [95% CI −0.5, −0.3] by acarbose, and −0.6 [−0.8, −0.4] by miglitol). Relative reductions in PPG by both drugs were similar for diabetic and non-diabetic individuals (43−54%). Acarbose and miglitol also significantly reduced mean PPI, with absolute and relative reductions being largest among individuals without diabetes.

**Conclusions:**

The present meta-analyses provide quantitative estimates of reductions of PPG and PPI responses by AGI drugs in diabetes and non-diabetic individuals. These data can serve as benchmarks for clinically relevant reductions in PPG and PPI via drug or diet and lifestyle interventions.

## Introduction

Elevated glucose levels in the postprandial state are a key feature of impaired glucose tolerance and diabetes^1^ and are a risk marker for cardiovascular diseases (CVD)^2^. Reducing exposure to high glucose levels in the postprandial state is therefore a target for diabetes patients, as emphasized in guidelines from the International Diabetes Federation, and the American Diabetes Association^3,4^. Increments in postprandial glucose (PPG) levels are generally considered as risk factors for micro and macrovascular complications via several pathways^5,6^. Therefore, PPG lowering is an important route for reducing chronic disease risk, also for the general population (at risk)^5^.

Direct evidence for the beneficial effects of reducing PPG comes from studies with alpha-glucosidase-inhibiting (AGI) drugs (acarbose, miglitol, voglibose). These drugs, which primarily act to lower PPG by slowing down the rate of carbohydrate digestion and gastrointestinal glucose uptake, have been shown to improve glycaemic control^7^ and reduce type 2 diabetes mellitus (T2DM) risk^8,9^. Long-term effects on CVD outcomes have been inconsistent^9,10^. In support of the benefits of reducing PPG, consumption of diets with a lower glycemic index (GI) or load have been associated with lower risk of coronary heart disease (CHD)^11^, and T2DM^12,13^. Furthermore, interventions with lower GI diets may improve glycaemic control and insulin sensitivity in particular in diabetes^14,15^.

Despite considerable literature supporting the likely health benefits of reducing PPG exposures, the size of PPG reduction that is needed to translate into a meaningful reduction in disease risk (factors) is currently unknown. Dietary trials with lower GI diets generally lack precise quantification of glycaemic exposure in real life^5^. AGI drugs have been approved for the treatment of diabetes since the 1990s^16^. These drugs have been studied for both acute postprandial effects and longer-term effects on risk factors. Meta-analyses of studies on effects of these AGIs have estimated these produce clinically relevant reductions of glycated haemoglobin (HbA1c) levels between −0.5 and −1.5% (5–16 mmol/mol) in subjects with diabetes^17–20^. Given the proven clinical efficacy of AGI drugs, their quantitative effects on PPG and postprandial insulin (PPI) could serve as a benchmark for interpreting the potential health relevance of the changes in PPG and PPI by other drugs or lifestyle interventions. However, to date, the acute postprandial effects of alpha-glucosidase inhibitors on glucose and insulin responses after a carbohydrate-containing meal have never been systematically reviewed and quantified.

Therefore, the aim of this review was to systematically review the quantitative effects of alpha-glucosidase inhibitors on acute PPG and PPI responses.

## Materials and methods

### Protocol, data source and search

PRISMA reporting guidelines were followed, and the protocol was registered in the International prospective register of systematic reviews (PROSPERO, number CRD42018085522). Electronic databases (Elsevier Medical Database (EMBASE) and the US National Library of Medicine database (MEDLINE via the PubMed portal) were used to search for relevant papers until March 13, 2018. The full search string can be found in Supplementary Data 1. The search was designed to identify controlled trials with AGI drugs together with a standardized carbohydrate load or mixed meal, studying effects on acute PPG and/or PPI responses.

### Selection of relevant studies

All titles and abstracts were screened in duplicate by two authors (M.A. and J.M.D.) and differences were resolved by consensus. Full-text publications were subjected to detailed examination against inclusion criteria by couples of two researchers (M.A., C.R., J.M.D., L.E., M.D.R.). Differences in the provisional inclusion or exclusion of studies by the two researchers were resolved by consultation. The following criteria were applied for inclusion: all populations, including healthy, pre-diabetes and diabetes (both type 1 and type 2 diabetes mellitus), use of AGI drug together with a standardized carbohydrate containing load, control treatment with placebo or no drug, and PPG or PPI as outcome. Exclusion criteria were not English language, oral glucose as carbohydrate load, no control treatment, co-intervention (an additional intervention in treatment arm but not the control arm), comparative study (with other drugs), and individuals who have undergone gastric surgery. Studies with insulin administration during the test meals were included for analyses of PPG outcomes, but excluded for analyses of insulin outcomes. Multiple arms of the same study were included when multiple arms were independent (had different control groups)^21^. Treatments arms with the same control group were included only if the arms were in different (subgroup) analyses.

### Data extraction and quality assessment

Data from included publications were extracted by one researcher (M.A.) and a random subsample of 10% was checked by another researcher (C.R.). Information on the study design, population, drug, dosage, test meal and outcome measures (glucose and insulin incremental, or total area under the curve (AUC), mean postprandial level, variance or *p*-values) were extracted. If no AUC or mean postprandial level was reported, postprandial response data per timepoint were extracted. Data from figures (i.e. bars for AUC, and responses per timepoint from graphs) were extracted using the Microsoft Excel add-in tool TM Image-to-data (tushar-mehta.com). If studies reported responses to multiple sequential meals, data from responses to the first meal were extracted. If the study duration was multiple days, data from day 1 were used.

The study quality was assessed with the Cochrane Collaboration’s tool for assessing risk of bias by scoring seven different items (random sequence generation, allocation concealment, blinding of participants and personnel, blinding of outcome data, incomplete outcome data, selective reporting, other bias)^21^. The item ‘blinding of outcome data’ was universally scored as ‘low risk’ because glucose and insulin are outcome measures than can be objectively assessed.

### Data synthesis and analysis

Glucose and insulin incremental or total AUCs, with variance measure, were transformed into SI units (mmol/l for glucose (=0.0555 mg/dl) and pmol/l for insulin (=6 microU/ml)). AUCs were divided by time (the duration of the postprandial measurement period) to express outcomes as average postprandial levels (for tAUC) or average postprandial increase (for iAUC). Standard errors (SE) were transformed into standard deviations (SD) (SE = SD/√*N*, where *N* = subject population). Where only *p*-values were reported, these were used to estimate the SE^21^. In cases where responses were only reported as data per timepoint (in Table or as a Figure), incremental AUC’s were calculated by the trapezoidal method as net incremental AUC^22^. The calculation of the variance of the iAUC was based on SDs of individual timepoints by using matrix algebra involving a covariance matrix with the assumed correlation structure being compound symmetry^23^. Assumed between-timepoints correlation were *r* = 0.75 for glucose and *r* = 0.5 for insulin. Meta-analysis was performed in Review Manager (RevMan) ([Computer program], version 5.3. Copenhagen: The Nordic Cochrane Centre, The Cochrane Collaboration, 2014). Random effects models with inverse variance weighing were used to estimate the combined absolute effects, which is expressed as a difference in average postprandial increase. Relative changes were calculated as 100% × (iAUC_drug_−iAUC_control_)/(iAUC_control_), with SE of relative change calculated as earlier described^24^, assuming a within subject correlation coefficient of 0.7. Heterogeneity and subgroup differences were assessed by *I*^2^ and Chi^2^ statistic (*p* < 0.05), respectively. Fixed effects models were run as sensitivity analyses.

Main analyses were conducted for each drug and in subgroups of dosage, for diabetes and non-diabetic individuals separately. Other subgroup analyses were performed if there were a minimum of three comparisons per subgroup, and these were: duration of postprandial measurement period, timing of drug administration (before or with meal), mixed meal versus carbohydrate meal (≤70 vs. >70% E), and carbohydrate content of the meal.

## Results

### Characteristics of included studies

**Fig. 1:**
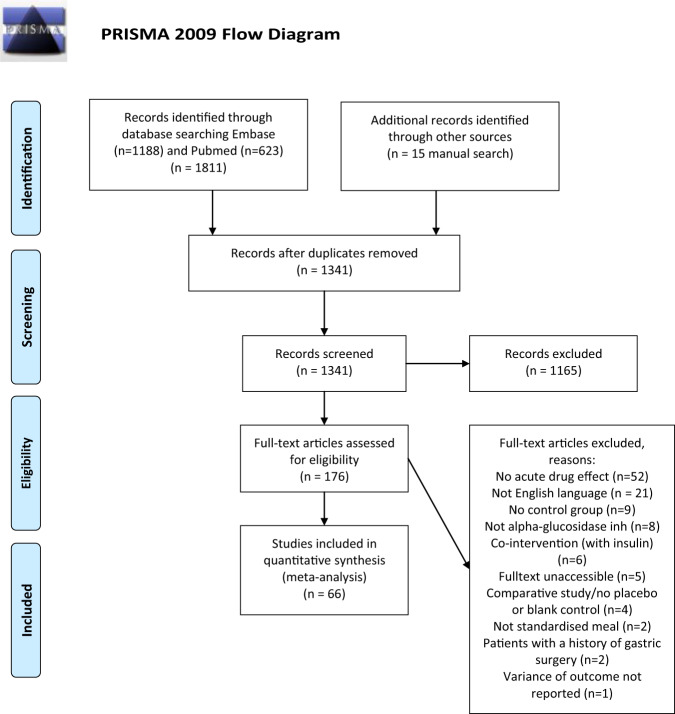
PRISMA flow diagram of literature search: PRISMA=preferred reporting items for systematic reviews and meta-analyses. PRISMA flow-diagram.

The search retrieved 1811 publications and an additional 15 potentially relevant publications were found manually and added to the database for screening (Fig. 1). After removal of duplicates, 1341 records were screened based on titles and abstracts; 176 full-text publications were finally assessed for eligibility.

The 66 included publications (Supplementary Table 1) comprised 134 comparisons, of which 127 comparisons had information on PPG and 106 comparisons had information on PPI. For PPG, the number of comparisons per drug was 64, 48, 6 and 8 for acarbose, miglitol, voglibose and emiglitate, respectively. For PPI, the number of comparisons per drug was 46, 42, 10 and 8 for acarbose, miglitol, voglibose and emiglitate, respectively. Most of the comparisons were among individuals without diabetes (84 out of 134) (Supplementary Table 1). Of the 50 comparisons among patients with diabetes, 6 were among type 1 diabetes and 44 among type 2 diabetes. The range in duration of postprandial measurement was 90–360 min, with a median and most frequent duration of 180 min. Standardized meals were mostly mixed meals (90 out of 127 comparisons), others were high carbohydrate loads (>70% E carbohydrates). Total energy content of standardized meals ranged from 200 to 900 kcal, and carbohydrate content ranged from 32 to 125 g (Supplementary Table 1).

### Acarbose and miglitol—main findings

Acarbose and miglitol significantly reduced mean PPG among individuals with and without diabetes (Table 1). The absolute effects of acarbose and miglitol on PPG were larger among individuals with diabetes (−1.5 mmol/l mean PPG [95% CI −1.9, −1.1] by acarbose, and −1.6 [−1.9, −1.4] by miglitol) as compared to individuals without diabetes (−0.4 mmol/l mean PPG [95% CI −0.5, −0.3] by acarbose, and −0.6 [−0.8, −0.4] by miglitol) (Chi^2^ for subgroup difference *p* < 0.05 for both acarbose and miglitol). Relative effects of both drugs on PPG were similar for diabetic and non-diabetic individuals with a percentage change by acarbose of −43% in both groups and by miglitol of −54% among individuals without diabetes and −50% among individuals with diabetes (Table 1).

**Table 1:** Absolute and relative effects of acarbose and miglitol on PPG and PPI by diabetes state by random effects model.

	Mean PPG (mmol/l) [95% CI]				Mean PPI (pmol/l) [95% CI]			
	*N*^a^	No diabetes	*N*^b^	Diabetes	*N*^a^	No diabetes	*N*^b^	Diabetes
*Acarbose*								
Mean difference, [95% CI]	30	−0.4 [−0.5, −0.3]	22	−1.5 [−1.9, −1.1]*	19	−66.7 [−97.1, −36.3]	15	−38.2 [−53.3, −-23.7]
Relative change, % [95% CI]	27	−43.3 [−51.2, −35.5]	19	−43.0 [−53.7, −32.3]	19	−64.9 [−76.1, −53.7]	15	−43.7 [−53.6, −33.9]*
*Miglitol*								
Mean difference, [95% CI]	16	−0.6 [−0.8, −0.4]	17	−1.6 [−1.9, −1.4]*	15	−68.6 [−95.2, −42.0]	13	−12.2 [−22.6, −1.7]*
Relative change, % [95% CI]	16	−54.3 [−62.9, −45.7]	17	−49.9 [−58.3, −41.4]	15	−77.5 [−106.5, −48.6]	13	−20.6 [−29.8, −11.5]*

*p < 0.05 for subgroup difference between healthy and diabetes by Chi^2^.

^a^*N* is the number of comparisons among non-diabetic individuals.

^b^*N* is the number of comparisons among diabetic individuals.

Acarbose and miglitol also significantly reduced mean PPI among individuals with diabetes (−38.2 pmol/l [95% CI −53.3, −23.2] by acarbose, and −12.2 [−22.6, −1.7] by miglitol) and without diabetes (−66.7 [−97.1, −36.3] by acarbose, and −68.6 [−95.2, −42.0] by miglitol (Table 1)). In contrast to effects seen on PPG, absolute and relative reductions on PPI by both drugs were generally larger among individuals without diabetes (Chi^2^ for subgroup difference *p* < 0.05 for all subgroup differences except for absolute reduction on PPI by acarbose (Table 1)).

In sensitivity analyses with fixed, instead of random effects models, results were largely similar to random effects model, except for a smaller effect of miglitol on PPI in healthy individuals (Supplementary Table 2).

### Acarbose and miglitol—subgroup analyses

Subgroup analyses with dose revealed that there were no differences between dosages for acarbose (based on studies in non-diabetics only) (Table 2 and Supplementary Fig. 1), but effects of miglitol on PPG were greater with increasing dose (Table 3 and Supplementary Fig. 1). There were no dose-effects of either drug on PPI. Further subgroup analyses showed that effects of acarbose on PPG were larger with a shorter measurement duration (120 min or less), among individuals without diabetes only (Table 2). Effects of miglitol on PPI were larger with shorter duration and were strongest when the drug was ingested before (as opposed to with) meal, among individuals with diabetes only (Table 3). Effects of miglitol on PPG were larger with lower carbohydrate content (<70 g), among individuals with diabetes only (Table 3). Finally, effects of miglitol on PPI were larger when consumed with high carbohydrate content (individuals without diabetes only) (Table 3).

**Table 2:** Subgroup analyses for acarbose effects on mean PPG and PPI stratified by diabetes state by random effects models.

	Mean PPG (mmol/l) [95% CI]				Mean PPI (pmol/l) [95% CI]			
	*N*^a^	No diabetes	*N*^b^	Diabetes	*N*^a^	No diabetes	*N*^b^	Diabetes
Overall	30	−0.4 [−0.5, −0.3]	22	−1.5 [−1.9, −1.1]*	19	−66.7 [−97.1, −36.3]	15	−38.2 [−53.3, −23.2]
Dose <100 mg	16	−0.6 [−0.7, −0.4]	–	–	9	−52.5 [−77.7, −27.3]	–	–
Dose 100 mg	12	−0.3 [−0.5, −0.1]	20	−1.5 [−1.9, −1.0]	12	−72.8 [−116.3, −29.3]	13	−37.9 [−53.8, −22.0]
Dose >100 mg	9	−0.3 [−0.5, −0.1]	–	–	4	−99.1 [−187.5, −10.6]	–	–
Duration 90–120 min	13	−0.6 [−0.7, −0.4]	7	−1.9 [−2.8, −1.0]	7	−54.9 [−83.2, −26.6]	3	−30.3 [−130.9, 70.4]
Duration 180–360 min	17	−0.2 [−0.3, −0.1]^#^	15	−1.2 [−1.5, −1.0]	12	−66.6 [−108.7, −24.5]	13	−34.7 [−47.5, −21.8]
Drug ingestion before meal	15	−0.4 [−0.5, −0.2]	7	−1.2 [−1.5, −0.9]	13	−57.8 [−80.6, −34.9]	6	−40.7 [−55.3, −26.0]
Drug ingestion with meal	15	−0.3 [−0.5, −0.2]	15	−1.6 [−2.2, −1.1]	6	−68.3 [−130.2, −6.5]	9	−37.6 [−66.0, −9.2]
Mixed meal	14	−0.5 [−0.7, −0.2]	21	−1.3 [−1.5, −1.2]	12	−67.7 [−97.5, −37.9]	12	−31.4 [−45.4, −17.4]
Carbohydrate meal	16	−0.3 [−0.4, −0.2]	3	−2.1 [−3.3, −1.0]	7	−61.0 [−115.1, −7.0]	3	−57.8 [−95.0, −20.7]
Carb content ≤median (70 g)	9	−0.3 [−0.5, −0.2]	10	−1.8 [−2.5, −1.1]	5	−53.8 [−85.4, −22.3]	10	−43.5 [−64.4, −22.6]
Carb content >median (70 g)	17	−0.4 [−0.5, −0.2]	6	−1.3 [−1.6, −1.0]	14	−69.3 [−108.7, −29.8]	5	−26.1 [−40.9, −11.4]

*p < 0.05 for subgroup difference between healthy and diabetes by Chi^2^; ^*#*^*p* < 0.05 for difference between subgroups by Chi^2^.

^a^*N* is the number of comparisons among non-diabetic individuals. Note that the number of comparisons in subgroups may exceed, or be lower than the number of comparisons for the overall estimate. For different reasons: (1) Multiple comparisons from the same study were excluded if they appeared in the same subgroup, but not if appearing in different subgroups; (2) For some comparisons, information was lacking to categorize to a specific subgroup (see Supplementary Table 1).

^b^*N* is the number of comparisons among diabetic individuals.

**Table 3:** Subgroup analyses for miglitol effects on mean PPG and PPI stratified by diabetes state by random effects models.

	Mean PPG (mmol/l) [95% CI]				Mean PPI (pmol/l) [95% CI]			
	*N*^a^	No diabetes	*N*^b^	Diabetes	*N*^a^	No diabetes	*N*^b^	Diabetes
Overall	16	−0.6 [−0.8, −0.4]	17	−1.6 [−1.9, −1.4]*	15	−68.6 [−95.2, −42.0]	13	−12.2 [−22.6, −1.7]*
Dose <50 mg	3	−0.2 [−0.5, 0.0]	–	–	3	−26.8 [−58.9, 5.3]	–	–
Dose 50 mg	12	−0.6 [−0.8, −0.4]	10	−1.5 [−1.8, −1.2]	11	−67.6 [−99.1, −36.1]	8	−30.2 [−54.7, −5.8]
Dose >50 mg	5	−0.8 [−1.1, −0.4]^#^	8	−2.1 [−2.4, −1.7]^#^	5	−67.0 [−85.6, −48.5]	7	−4.8 [−11.8, 2.1]
Duration 120–180 min	13	−0.7 [−0.9, −0.4]	7	−1.4 [−1.7, −1.1]	12	−70.6 [−103.3, −37.9]	5	−50.1 [−72.1, −28.2]
Duration 210–300 min	3	−0.6 [−0.9, −0.2]	12	−1.9 [−2.3, −1.5]	3	−64.2 [−87.5, −40.9]	8	−3.3 [−10.1, 3.5]^#^
Drug ingestion before meal	12	−0.6 [−0.8, −0.4]	7	−1.5 [−1.8, −1.2]	11	−74.8 [−104.8, −44.9]	5	−54.4 [−78.3, −30.5]
Drug ingestion with meal	4	−0.7 [−1.3, −0.1]	8	−1.7 [−2.3, −1.2]	4	−50.9 [−98.4, −3.4]	6	−11.9 [−24.9, 1.1]^#^
Mixed meal	10	−0.6 [−0.9, −0.4]	16	−1.7 [−1.9, −1.4]	9	−89.4 [−123.9, −54.9]	12	−11.9 [−22.7, −1.1]
Carbohydrate meal	6	−0.7 [−1.1, −0.3]	–	–	6	−45.3 [−76.3, −14.3]	–	–
Carb content ≤median (70 g)	6	−0.5 [−0.7, −0.2]	12	−1.8 [−2.2, −1.5]	6	−38.5 [−64.8, −12.2]	8	−6.4 [−16.0, 3.3]
Carb content >median (70 g)	10	−0.8 [−1.0, −0.5]	4	−1.3 [−1.6, −1.0]^#^	9	−103.5 [−140.7, −66.3]^#^	4	−31.4 [−58.4, −4.4]

*p < 0.05 for subgroup difference between healthy and diabetes by Chi^2^; ^#^*p* < 0.05 for difference between subgroups by Chi^2^.

^a^*N* is the number of comparisons among non-diabetic individuals. Note that the number of comparisons in subgroups may exceed, or be lower than the number of comparisons for the overall estimate. For different reasons: (1) Multiple comparisons from the same study were excluded if they appeared in the same subgroup, but not if appearing in different subgroups; (2) For some comparisons, information was lacking to categorize to a specific subgroup (see Supplementary Table 1).

^b^*N* is the number of comparisons among diabetic individuals.

### Voglibose and emiglitate

The overall effect of voglibose on PPG and PPI (based on four and five comparisons in predominantly non-diabetic individuals) was not significant (mean difference [95% CI] −0.3 [−0.6, 0.01] and −30.8 [−76.9, 15.3] for PPG and PPI, respectively). The overall effect of emiglitate (based on four comparisons each) was a reduction in PPG and PPI (mean difference [95% CI] −0.3 [−0.5, −0.03] and −44.3 [−66.1, −22.5] for PPG and PPI, respectively) (Forest plots with study quality assessments are given in Supplementary Fig. 1).

### Study quality assessment

The risk of bias was low for items blinding of outcome data, incomplete outcome data, selective reporting and other biases most studies. However, the information about the quality items randomization, allocation concealment, and blinding of participants and personnel was lacking or incomplete in most studies (Supplementary Fig. 1).

## Discussion

This is the first systematic investigation aiming to quantify the effects of AGI drugs on acute PPG and PPI responses. As expected, AGI drugs reduce PPG and PPI responses among individuals with and without diabetes, with reductions in incremental PPG of ~45–50% and of ~20–75% in incremental PPI. Absolute effects on PPG were largest among diabetes patients, but relative effects were comparable for individuals with and without diabetes. Absolute and relative effects on PPI were larger among individuals without diabetes. Effects of acarbose were not dose-dependent, but effects of miglitol on PPG were larger with increasing dose.

The effects of AGI drugs on PPG and PPI are well known from their primary mechanism of action, which is the slowing down of carbohydrate digestion^16^. The present meta-analysis shows a larger absolute effect of acarbose and miglitol on PPG among individuals with versus without diabetes, most likely because the higher overall PPG response in the former. Indeed, while absolute reductions in PPG were larger in that group, relative reductions were comparable between individuals with and without diabetes. Previous studies do not provide strong indications that differences in rates of absorption contribute to the greater absolute effects of AGI’s in diabetes. Indeed, faster, but also slower or equal, rates of gastric emptying have been observed in obesity and diabetes, and data on this are not conclusive^25^. Data from a stable isotope study show comparable rates of glucose absorption in individuals with and without diabetes^26^.

In contrast to the stronger absolute effects on PPG among individuals with diabetes, the reduction in PPI was largest among non-diabetic individuals. Patterns of insulin secretion in diabetes patients have been shown to be more irregular and not closely linked to glucose responses^27^. The reduction of PPI in healthy volunteers is probably the result of the rapid adaptation of insulin secretion to reduced PPG responses, which is not optimal in diabetes patients.

An overall dose–response relationship with PPG lowering was observed for miglitol but not acarbose (based on studies in non-diabetics only). The lack of a dose–response effect for acarbose is in line with the within-study effects of underlying studies; most of the included studies do not provide indications for dose–response effects^28–35^. In meta-analyses of longer-term studies with acarbose in diabetes, there was no dose-dependent effect seen on HbA1c^17,19^, but there was for post-load glucose^17^. The observed dose–response effect for miglitol on PPG is in line with the within-study comparisons in underlying studies, most though not all of which indicate dose-dependent effects of miglitol on PPG^36–39^. Moreover, longer-term studies, although scarce, have been suggestive of a dose–response effect of miglitol on HbA1c^17^.

Effects of AGI drugs on responses to mixed meals vs. carbohydrate meals, and high vs. low carbohydrate loads, did not provide support for the hypothesis that AGIs are most effective when accompanying high carbohydrate meals. Asian diets are generally higher in carbohydrate content, but a meta-analysis on effects of AGIs in Asian versus non-Asian populations did not reveal differential effects on glycaemic control^20^. However, we cannot fully rule out differential effects dependent on meal composition because the studies with high carbohydrate content were most often mixed meals, and the number of studies was too small to differentiate for both meal composition and carbohydrate content.

Our analysis indicates that AGI drugs under standardized acute conditions reduce incremental PPG and PPI by ~45–50% and ~20–75%, respectively. Based on previous studies on the longer-term effects of use of AGIs, achievement of such acute changes under standardized conditions can be expected to lead to clinically relevant chronic effects over time. Previous meta-analyses of studies with AGIs have estimated clinically relevant reductions of HbA1c between −0.5 and −1.5% in subjects with diabetes^17–20^. Moreover, the limited number of long-term studies (of 1 year or longer) with AGIs in individuals at increased risk for diabetes is indicative of a reduction in risk of development of overt diabetes^9,40^. However, clinically relevant effects on PPG and PPI should always be considered in the context of the mechanisms of action of the intervention and the metabolic effects beyond PPG and PPI. AGIs as a class reduces carbohydrate digestion, glycaemic variability, lipids, blood pressure, coagulation factors, and have impact on incretin hormones and gut microbiota^16^. Other diet, lifestyle or drug intervention that lower PPG, will also impact a range of upstream and downstream effects, which should be considered when estimating longer-term effects on risk factors or disease outcomes.

The reduction of incremental PPG of ~45–50%, or 0.5 mmol/l mean PPG (non-diabetes) and 1.5 mmol/l (in diabetes) as achieved by AGIs may be regarded as a point of reference, rather than a threshold per se. Lower reductions in PPG might lead to relevant changes in metabolic risk factors over time, depending on the related metabolic effects. Indeed, low glycaemic index diets exert improvements in glycaemic control and insulin sensitivity^14,15^, while the reductions in PPG and PPI achieved by such diets may be smaller than the effects of drugs. However, it should be noted that a direct quantification of diets effects on PPG and PPI is largely lacking^5^.

A strength of this study is the comprehensive set of studies included because data from all studies identified as eligible according to inclusion and exclusion criteria were extracted and included in the analyses. A limitation is the lack of standardization in PPG and PPI responses, as different studies have applied different measurement durations and higher or lower frequency of measurements during the postprandial response. This may have led to a greater uncertainty around effect sizes and hence reduced the statistical power to identify effects and differences in effects between subgroups. In addition, the accurate assessment of the PPG/PPI response is ultimately related to the number of time points included in the individual studies. Two of the papers^29,41^ included in our analysis were from studies with only three time points; however, the response to these interventions was within the range reported of other studies.

In summary, the present meta-analyses provide quantitative estimates of effects of AGI drugs on PPG and PPI responses. Absolute reductions in PPG are larger among individuals with diabetes, but reductions in PPI are larger among non-diabetic individuals. These data can serve as benchmarks for clinically relevant reductions in PPG and PPI via drug or diet and lifestyle interventions.

## Supplementary information

Supplementary Information
