# Supplementary material for: Effects of alpha-glucosidase-inhibiting drugs on acute postprandial glucose and insulin responses: a systematic review and meta-analysis
Source: Nutr Diabetes. 2021 Mar 3;11:11. doi: 10.1038/s41387-021-00152-5 (PMC7930031; doi:10.1038/s41387-021-00152-5)
Supplement: Supplementary file 1 — Supplementary Information [file 41387_2021_152_MOESM1_ESM.pdf]

Effects of Alpha-Glucosidase Inhibiting Drugs on Acute Postprandial Glucose and Insulin Responses: a Systematic Review and Meta-Analysis

Supplementary Information

Supplementary Figure 1:

Study or Subgroup	Acarbose Mean [mmol/l]	Acarbose SD [mmol/l]	Acarbose Total	Control Mean [mmol/l]	Control SD [mmol/l]	Control Total	Weight	Mean Difference IV, Random, 95% CI [mmol/l]
**5.1.1 Dose <100 mg**								
Aziz 2013 (1)	5.606	0.467	13	5.629	0.234	13	3.1%	-0.02 [-0.31, 0.26]
Debeljak 2016	0.502	0.47	31	0.884	0.555	31	3.3%	-0.38 [-0.64, -0.13]
Hillebrand 1979a (1)	0.584	0.669	12	0.929	0.62	12	2.1%	-0.35 [-0.86, 0.17]
Hillebrand 1979b (1)	0.449	0.477	24	0.971	0.602	24	3.0%	-0.52 [-0.83, -0.21]
Jenkins 1979 (1)	5.2	0.3	8	6.1	0.4	8	2.9%	-0.90 [-1.25, -0.55]
Jenkins 1979 (2)	4.7	0.3	8	5.5	0.4	8	2.9%	-0.80 [-1.15, -0.45]
Kageyama 1997 (1)	5.286	0.633	20	5.644	0.757	20	2.5%	-0.36 [-0.79, 0.07]
Koytchev 2009 (1)	0.343	0.254	12	0.718	0.757	12	2.4%	-0.37 [-0.83, 0.08]
Koytchev 2009 (3)	0.136	0.231	12	0.843	0.599	12	2.8%	-0.71 [-1.07, -0.34]
O'Dea 1985 (1)	0.407	0.188	6	0.74	0.188	6	3.5%	-0.33 [-0.55, -0.12]
O'Dea 1985 (2)	0.227	0.147	6	0.74	0.188	6		Not estimable
O'Dea 1985 (3)	0.147	0.041	6	0.74	0.188	6		Not estimable
Taylor 1982 (1)	0.244	0.269	4	1.389	0.531	4	1.9%	-1.15 [-1.73, -0.56]
Taylor 1982 (2)	0.266	0.225	4	1.018	0.297	4	2.8%	-0.75 [-1.12, -0.39]
Taylor 1982 (3)	0.156	0.487	4	0.592	0.325	4	1.9%	-0.44 [-1.01, 0.14]
Wachters-Hagedoorn 2007	0.214	0.157	7	0.544	0.381	7	3.1%	-0.33 [-0.64, -0.02]
Yuhong 2014	5.46	2.668	18	5.463	2.728	18	0.4%	-0.00 [-1.77, 1.76]
Zulkarnain 2009	0.69	0.48	12	2	0.42	12	2.8%	-1.31 [-1.67, -0.95]
**Subtotal (95% CI)**			**195**			**195**	**41.3%**	**-0.56 [-0.73, -0.39]**
Heterogeneity: Tau² = 0.08; Chi² = 51.64, df = 15 (P < 0.00001); I² = 71%								
Test for overall effect: Z = 6.40 (P < 0.00001)								
**5.1.2 Dose 100 mg**								
Arakawa 2008 (2)	6.718	0.983	24	6.878	0.914	24	2.1%	-0.16 [-0.70, 0.38]
Aziz 2013 (2)	4.578	0.187	13	5.629	0.234	13	3.6%	-1.05 [-1.21, -0.89]
Dimitriadis 1982	0.345	0.163	6	0.349	0.239	6	3.4%	-0.00 [-0.24, 0.23]
Enc 2001 (1)	5.134	0.768	14	4.64	0.685	14	2.1%	0.49 [-0.05, 1.03]
Enc 2001 (2)	4.09	0.231	12	4.446	0.846	12	2.2%	-0.36 [-0.85, 0.14]
Gentilcore 2005	1.166	0.547	8	1.24	0.797	8	1.6%	-0.07 [-0.74, 0.60]
Hillebrand 1979b (2)	0.546	0.482	24	0.971	0.602	17	2.9%	-0.42 [-0.77, -0.08]
Hopfner 1997 (1)	0.39	0.148	24	0.593	0.156	24	3.9%	-0.20 [-0.29, -0.12]
Hopfner 1997 (2)	0.32	0.163	24	0.523	0.175	24	3.8%	-0.20 [-0.30, -0.11]
Kageyama 1997 (2)	5.414	0.558	20	5.644	0.757	20	2.6%	-0.23 [-0.64, 0.18]
Kageyama 2000 (1)	6.263	0.555	12	6.666	0.74	12	2.1%	-0.40 [-0.93, 0.12]
Shibao 2007	0.549	0.278	13	1.075	0.377	13	3.3%	-0.53 [-0.78, -0.27]
**Subtotal (95% CI)**			**194**			**187**	**33.5%**	**-0.29 [-0.50, -0.09]**
Heterogeneity: Tau² = 0.10; Chi² = 109.70, df = 11 (P < 0.00001); I² = 90%								
Test for overall effect: Z = 2.76 (P = 0.006)								
**5.1.3 Dose >100 mg**								
Caspary 1978	0.947	0.532	7	0.993	0.413	7	2.2%	-0.05 [-0.54, 0.45]
Frati Munari 1998 (2)	0.233	0.211	10	0.333	0.105	10	3.7%	-0.10 [-0.25, 0.05]
Hillebrand 1979a (2)	0.522	0.625	12	0.929	0.62	12	2.2%	-0.41 [-0.91, 0.09]
Hillebrand 1979a (3)	0.36	0.597	12	0.929	0.62	12		Not estimable
Hillebrand 1979b (3)	0.539	0.369	24	0.971	0.602	24	3.2%	-0.43 [-0.71, -0.15]
Koytchev 2009 (2)	0.037	0.17	12	0.718	0.757	12	2.5%	-0.68 [-1.12, -0.24]
Koytchev 2009 (4)	0.072	0.286	12	0.843	0.599	12	2.7%	-0.77 [-1.15, -0.40]
Lee 2012 (1)	5.994	0.636	5	6.47	0.398	5	1.7%	-0.48 [-1.13, 0.18]
Lee 2012 (2)	0.618	0.347	33	0.631	0.438	33	3.5%	-0.01 [-0.20, 0.18]
Lee 2012 (3)	0.467	0.287	33	0.77	0.539	33	3.5%	-0.30 [-0.51, -0.09]
**Subtotal (95% CI)**			**148**			**148**	**25.2%**	**-0.32 [-0.49, -0.14]**
Heterogeneity: Tau² = 0.04; Chi² = 23.99, df = 8 (P = 0.002); I² = 67%								
Test for overall effect: Z = 3.59 (P = 0.0003)								
**Total (95% CI)**			**537**			**530**	**100.0%**	**-0.42 [-0.53, -0.30]**
Heterogeneity: Tau² = 0.08; Chi² = 209.90, df = 36 (P < 0.00001); I² = 83%								
Test for overall effect: Z = 7.30 (P < 0.00001)								
Test for subgroup differences: Chi² = 5.27, df = 2 (P = 0.07), I² = 62.0%								

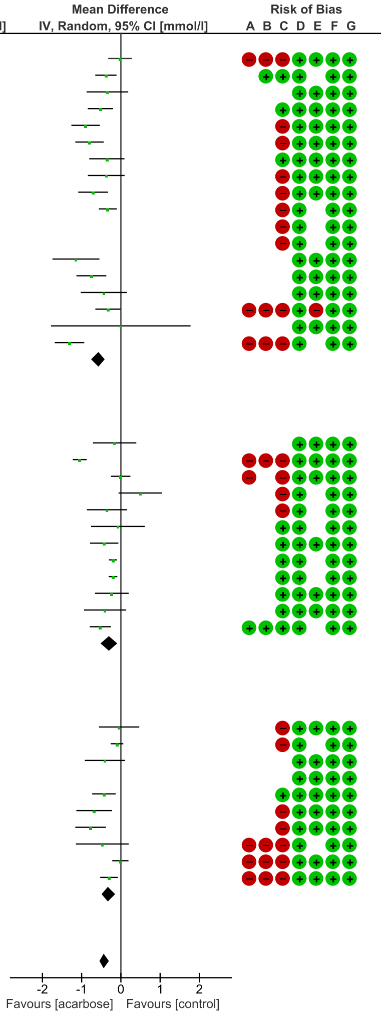

Risk of bias legend
(**A**) Random sequence generation (selection bias)
(**B**) Allocation concealment (selection bias)
(**C**) Blinding of participants and personnel (performance bias)
(**D**) Blinding of outcome assessment (detection bias)
(**E**) Incomplete outcome data (attrition bias)
(**F**) Selective reporting (reporting bias)
(**G**) Other bias

Supplementary Figure 1a. Forest plot of acarbose studies on mean postprandial glucose response levels in individuals without diabetes.

Study or Subgroup	Acarbose Mean [mmol/l]	Acarbose SD [mmol/l]	Acarbose Total	Control Mean [mmol/l]	Control SD [mmol/l]	Control Total	Weight	Mean Difference IV, Random, 95% CI [mmol/l]
**6.1.1 Dose 100 mg**								
Ceriello 1996	1.377	1.576	17	3.083	2.316	17	4.4%	-1.71 [-3.04, -0.37]
Chen 2016	1.818	0.257	12	4.738	0.36	12	7.4%	-2.92 [-3.17, -2.67]
DeLeon 2002 (3)	4.224	1.193	10	5.669	1.79	10	4.4%	-1.44 [-2.78, -0.11]
Frank 1998 (1)	-0.673	2.169	20	2.336	1.907	20	4.6%	-3.01 [-4.27, -1.74]
Frank 1998 (2)	-1.877	3.338	20	-0.118	3.084	20	2.9%	-1.76 [-3.75, 0.23]
Hermanns 2004	15.708	2.445	30	17.918	4.125	30	3.5%	-2.21 [-3.93, -0.49]
Hucking 2005	0.409	0.547	10	1.587	1.125	10	6.1%	-1.18 [-1.95, -0.40]
Kado 1998	3.865	0.964	20	5.126	1.239	20	6.4%	-1.26 [-1.95, -0.57]
Kusunoki 2013	5.411	1.116	10	6.299	1.532	10	4.9%	-0.89 [-2.06, 0.29]
Lettieri 1998	11.68	0.574	37	13.13	0.574	37	7.4%	-1.45 [-1.71, -1.19]
Ogawa 2004	1.873	1.314	40	2.86	1.524	40	6.6%	-0.99 [-1.61, -0.36]
Rabasa-Lhoret 2001	1.187	1.749	7	2.886	1.57	7	3.4%	-1.70 [-3.44, 0.04]
Rosak 2002 (1)	1.397	0.863	22	2.669	1.344	22	6.5%	-1.27 [-1.94, -0.60]
Rosak 2002 (2)	-0.987	0.473	21	-0.051	1.169	21	6.8%	-0.94 [-1.48, -0.40]
Seifarth 1998	11.325	2.349	11	12.083	2.405	11	2.9%	-0.76 [-2.74, 1.23]
Shimabukuro 2006	1.538	0.967	14	2.233	1.122	14	6.1%	-0.70 [-1.47, 0.08]
Tamasawa 2015	10.129	3.169	16	10.534	3.269	16	2.5%	-0.41 [-2.64, 1.83]
Ueno 2015 (2)	3.099	0.455	11	4.388	0.513	11	7.1%	-1.29 [-1.69, -0.88]
Uttenthal 1986	2.725	1.63	17	4.244	2.099	17	4.6%	-1.52 [-2.78, -0.26]
Walton 1979	-0.041	4.193	7	1.797	2.685	7	1.2%	-1.84 [-5.53, 1.85]
**Subtotal (95% CI)**			**352**			**352**	**100.0%**	**-1.46 [-1.89, -1.02]**

Heterogeneity: Tau² = 0.64; Chi² = 130.30, df = 19 (P < 0.00001); I² = 85%
Test for overall effect: Z = 6.58 (P < 0.00001)

**Total (95% CI)**			**352**			**352**	**100.0%**	**-1.46 [-1.89, -1.02]**

Heterogeneity: Tau² = 0.64; Chi² = 130.30, df = 19 (P < 0.00001); I² = 85%
Test for overall effect: Z = 6.58 (P < 0.00001)
Test for subgroup differences: Not applicable

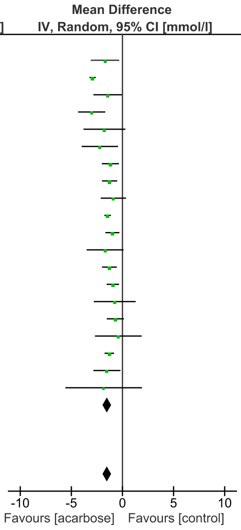

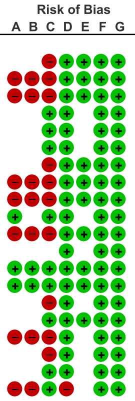

Risk of bias legend
(**A**) Random sequence generation (selection bias)
(**B**) Allocation concealment (selection bias)
(**C**) Blinding of participants and personnel (performance bias)
(**D**) Blinding of outcome assessment (detection bias)
(**E**) Incomplete outcome data (attrition bias)
(**F**) Selective reporting (reporting bias)
(**G**) Other bias

Supplementary Figure 1b. Forest plot of acarbose studies on mean postprandial glucose response levels in individuals with diabetes.

Study or Subgroup	Acarbose Mean [pmol/l]	Acarbose SD [pmol/l]	Acarbose Total	Control Mean [pmol/l]	Control SD [pmol/l]	Control Total	Weight	Mean Difference IV, Random, 95% CI [pmol/l]
**5.2.1 Dose <100 mg**								
Hillebrand 1979a (1)	194.43	58.45	12	381.4	125.98	12	3.4%	-186.97 [-265.55, -108.39]
Hillebrand 1979b (1)	119.53	99.91	24	231.7	112.51	24	3.9%	-112.17 [-172.37, -51.97]
Jenkins 1979 (1)	195	82.71	8	250.63	97.36	8	3.1%	-55.63 [-144.15, 32.89]
Jenkins 1979 (2)	116.88	26.46	8	151.94	38.18	8	4.7%	-35.06 [-67.25, -2.87]
Kageyama 1997 (1)	219.9	112.7	20	321.3	171.73	20	3.1%	-101.40 [-191.42, -11.38]
O'Dea 1985 (1)	47.6	17.15	6	97.6	25.47	6	4.9%	-50.00 [-74.57, -25.43]
O'Dea 1985 (2)	24.6	5.54	6	97.6	25.47	6		Not estimable
O'Dea 1985 (3)	10.8	7.35	6	97.6	25.47	6		Not estimable
Uttenthal 1987 (1)	11.46	6.11	5	18.63	12.95	5	5.1%	-7.17 [-19.72, 5.38]
Wachters-Hagedoorn 2007	7.65	6.22	7	29.9	16.8	7	5.1%	-22.25 [-35.52, -8.98]
Yuhong 2014	206.9	455.17	18	275.94	563.49	18	0.5%	-69.04 [-403.67, 265.59]
**Subtotal (95% CI)**			**108**			**108**	**33.7%**	**-52.50 [-77.67, -27.34]**
Heterogeneity: Tau² = 800.59; Chi² = 39.89, df = 8 (P < 0.00001); I² = 80%								
Test for overall effect: Z = 4.09 (P < 0.0001)								
**5.2.2 Dose 100 mg**								
Arakawa 2008 (2)	169.83	63.8	24	222.43	86.2	24	4.4%	-52.60 [-95.51, -9.69]
Dimitriadis 1982	156.86	53.69	6	181.82	62.04	6	3.8%	-24.96 [-90.61, 40.69]
Enc 2001 (1)	74.4	24.69	14	142.8	51.63	14	4.8%	-68.40 [-98.38, -38.42]
Enc 2001 (2)	85.8	87.3	12	204.6	164.2	12	2.7%	-118.80 [-224.02, -13.58]
Gentilcore 2005	74.14	5.88	8	234.18	17.4	8	5.1%	-160.04 [-172.77, -147.31]
Hillebrand 1979b (2)	87.78	90.02	24	231.7	112.51	24	4.0%	-143.92 [-201.57, -86.27]
Hopfner 1997 (1)	11.64	24.68	24	24.38	25.58	24	5.0%	-12.74 [-26.96, 1.48]
Hopfner 1997 (2)	9.84	26.85	24	29.44	29	24	5.0%	-19.60 [-35.41, -3.79]
Kageyama 1997 (2)	204.9	112.7	20	321.3	171.73	20	3.1%	-116.40 [-206.42, -26.38]
Kageyama 2000 (1)	290.1	125.95	12	406.8	143.41	12	2.6%	-116.70 [-224.69, -8.71]
Shibao 2007	54.09	33.88	13	131.64	78.58	13	4.3%	-77.55 [-124.07, -31.03]
Uttenthal 1987 (2)	12.16	10.05	5	18.63	12.95	5	5.0%	-6.47 [-20.84, 7.90]
**Subtotal (95% CI)**			**186**			**186**	**49.8%**	**-72.80 [-116.30, -29.29]**
Heterogeneity: Tau² = 5115.59; Chi² = 372.11, df = 11 (P < 0.00001); I² = 97%								
Test for overall effect: Z = 3.28 (P = 0.001)								
**5.2.3 Dose > 100 mg**								
Frati Munari 1998 (2)	14.57	23.19	10	79.6	98.03	10	3.9%	-65.03 [-127.47, -2.59]
Hillebrand 1979a (2)	204.23	60.65	12	381.4	125.98	12	3.4%	-177.17 [-256.28, -98.06]
Hillebrand 1979a (3)	140.48	48.21	12	381.4	125.98	12		Not estimable
Hillebrand 1979b (3)	75.88	61.87	24	231.7	112.51	24	4.2%	-155.82 [-207.19, -104.45]
Uttenthal 1987 (3)	5.9	4.34	5	18.63	12.95	5	5.1%	-12.73 [-24.70, -0.76]
Uttenthal 1987 (4)	5.9	2.96	5	18.63	12.95	5		Not estimable
**Subtotal (95% CI)**			**51**			**51**	**16.5%**	**-99.07 [-187.53, -10.61]**
Heterogeneity: Tau² = 7346.94; Chi² = 44.74, df = 3 (P < 0.00001); I² = 93%								
Test for overall effect: Z = 2.20 (P = 0.03)								
**Total (95% CI)**			**345**			**345**	**100.0%**	**-72.63 [-97.38, -47.88]**

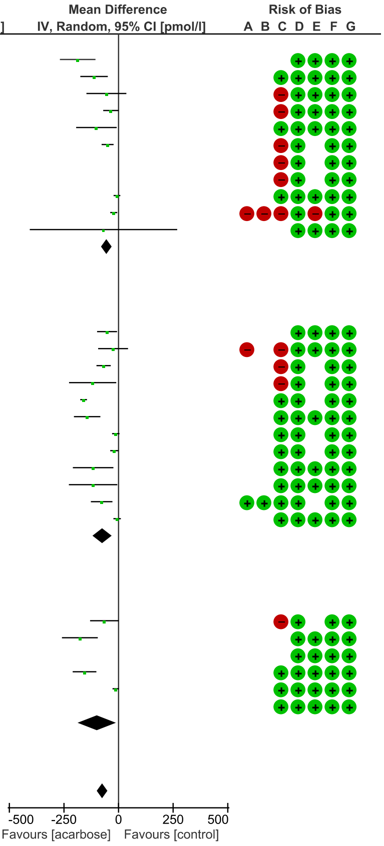

Heterogeneity: Tau² = 3109.95; Chi² = 514.44, df = 24 (P < 0.00001); I² = 95%
Test for overall effect: Z = 5.75 (P < 0.00001)
Test for subgroup differences: Chi² = 1.42, df = 2 (P = 0.49), I² = 0%

Risk of bias legend
(**A**) Random sequence generation (selection bias)
(**B**) Allocation concealment (selection bias)
(**C**) Blinding of participants and personnel (performance bias)
(**D**) Blinding of outcome assessment (detection bias)
(**E**) Incomplete outcome data (attrition bias)
(**F**) Selective reporting (reporting bias)
(**G**) Other bias

Supplementary Figure 1c. Forest plot of acarbose studies on mean postprandial insulin response levels in individuals without diabetes.

Study or Subgroup	Acarbose Mean [pmol/l]	Acarbose SD [pmol/l]	Acarbose Total	Control Mean [pmol/l]	Control SD [pmol/l]	Control Total	Weight	Mean Difference IV, Random, 95% CI [pmol/l]
**6.2.1 Dose 100 mg**								
Ceriello 1996	39.99	35.03	17	67.2	43.35	17	9.6%	-27.21 [-53.70, -0.72]
Chen 2016	126.06	26.23	12	218.34	40.34	12	9.5%	-92.28 [-119.50, -65.06]
DeLeon 2002 (3)	147.3	64.51	10	162.9	92.02	10	3.7%	-15.60 [-85.25, 54.05]
Hucking 2005	37.95	24.14	10	57.07	24.35	10	10.7%	-19.12 [-40.37, 2.13]
Kado 1998	74.96	30.94	20	103.83	51.8	20	9.7%	-28.87 [-55.31, -2.43]
Kusunoki 2013	72.2	27.2	10	92.2	39.4	10	9.0%	-20.00 [-49.67, 9.67]
Ogawa 2004	46.33	40.98	40	61.74	44.03	40	11.2%	-15.41 [-34.05, 3.23]
Rosak 2002 (1)	238.5	115.65	22	339	249.9	22	1.7%	-100.50 [-215.56, 14.56]
Rosak 2002 (2)	365.25	241.65	21	575.1	323.7	21	0.8%	-209.85 [-382.62, -37.08]
Seifarth 1998	85.28	49.75	11	101.39	56.2	11	6.5%	-16.11 [-60.46, 28.24]
Shimabukuro 2006	107.08	18.33	14	170	33.75	14	10.9%	-62.92 [-83.04, -42.80]
Ueno 2015 (2)	129.5	31.36	11	167.25	42.97	11	8.7%	-37.75 [-69.19, -6.31]
Uttenthal 1986	63.73	44.19	17	101.18	58.91	17	8.0%	-37.45 [-72.46, -2.44]
**Subtotal (95% CI)**			**215**			**215**	**100.0%**	**-37.88 [-53.80, -21.95]**
Heterogeneity: Tau² = 501.48; Chi² = 38.44, df = 12 (P = 0.0001); I² = 69%								
Test for overall effect: Z = 4.66 (P < 0.00001)								
**Total (95% CI)**			**215**			**215**	**100.0%**	**-37.88 [-53.80, -21.95]**

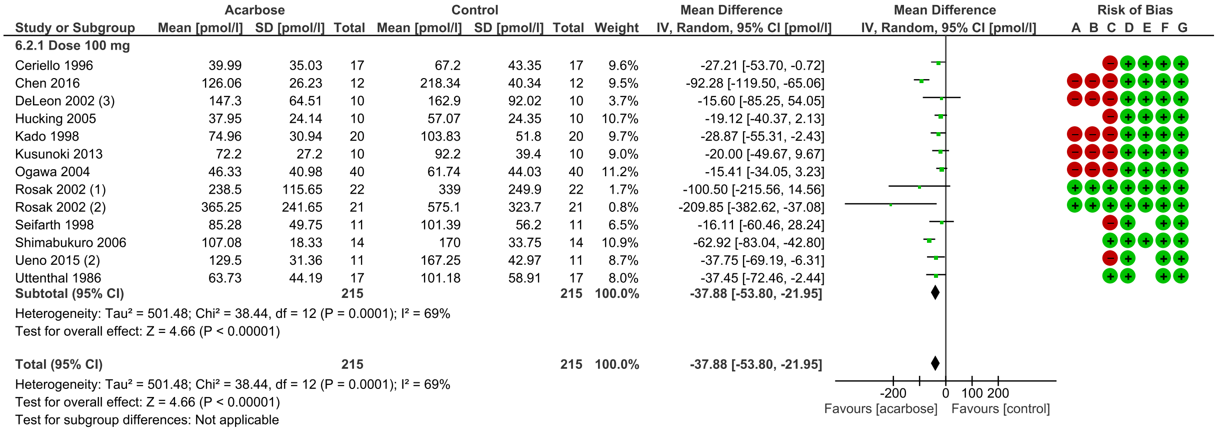

Heterogeneity: Tau² = 501.48; Chi² = 38.44, df = 12 (P = 0.0001); I² = 69%
Test for overall effect: Z = 4.66 (P < 0.00001)
Test for subgroup differences: Not applicable

Risk of bias legend
(**A**) Random sequence generation (selection bias)
(**B**) Allocation concealment (selection bias)
(**C**) Blinding of participants and personnel (performance bias)
(**D**) Blinding of outcome assessment (detection bias)
(**E**) Incomplete outcome data (attrition bias)
(**F**) Selective reporting (reporting bias)
(**G**) Other bias

Supplementary Figure 1d. Forest plot of acarbose studies on mean postprandial insulin response levels in individuals with diabetes.

Study or Subgroup	Miglitol Mean [mmol/l]	Miglitol SD [mmol/l]	Miglitol Total	Control Mean [mmol/l]	Control SD [mmol/l]	Control Total	Weight	Mean Difference IV, Random, 95% CI [mmol/l]
**7.1.1 Dose <50 mg**								
Kennedy 1987 (1)	0.783	0.518	8	1.127	0.549	8	4.8%	-0.34 [-0.87, 0.18]
Lembcke 1991a (1)	0.492	0.709	8	0.714	0.58	8	3.8%	-0.22 [-0.86, 0.41]
Lembcke 1991a (4)	0.527	0.405	8	0.73	0.335	8	6.7%	-0.20 [-0.57, 0.16]
**Subtotal (95% CI)**			**24**			**24**	**15.4%**	**-0.24 [-0.51, 0.03]**
Heterogeneity: Tau² = 0.00; Chi² = 0.19, df = 2 (P = 0.91); I² = 0%								
Test for overall effect: Z = 1.77 (P = 0.08)								
**7.1.2 Dose 50 mg**								
Aoki 2007b (1)	0.538	0.579	15	0.813	0.463	15	6.6%	-0.27 [-0.65, 0.10]
Aoki 2007b (2)	0.574	0.431	15	0.813	0.463	15		Not estimable
Aoki 2007b (3)	0.353	0.306	15	0.813	0.463	15		Not estimable
Aoki 2010 (1)	5.645	0.543	10	6.58	0.723	10	4.5%	-0.94 [-1.50, -0.37]
Aoki 2010 (2)	5.455	0.301	10	5.817	0.965	10	3.9%	-0.36 [-0.99, 0.26]
Aoki 2014 (1)	5.853	0.533	11	6.966	1.171	11	3.0%	-1.11 [-1.87, -0.35]
Aoki 2014 (2)	5.254	0.526	11	6.323	1.279	11	2.7%	-1.07 [-1.89, -0.25]
Arakawa 2008 (1)	6.67	1.028	24	6.878	0.914	24	4.5%	-0.21 [-0.76, 0.34]
Joubert 1985	0.443	0.363	12	1.227	0.863	12	4.7%	-0.78 [-1.31, -0.25]
Kaku 2012	0.519	0.309	20	1.156	0.572	20	7.9%	-0.64 [-0.92, -0.35]
Kennedy 1987 (2)	0.966	0.487	8	1.132	0.816	8	3.6%	-0.17 [-0.82, 0.49]
Lembcke 1991a (2)	0.488	0.761	8	0.714	0.58	8	3.6%	-0.23 [-0.89, 0.44]
Lembcke 1991a (5)	0.435	0.269	8	0.73	0.335	8	7.7%	-0.29 [-0.59, 0.00]
Masuda 2011 (1)	6.42	0.677	10	7.849	0.828	10	3.6%	-1.43 [-2.09, -0.77]
Masuda 2011 (2)	7.043	0.742	10	7.849	0.828	10		Not estimable
**Subtotal (95% CI)**			**147**			**147**	**56.3%**	**-0.57 [-0.78, -0.36]**
Heterogeneity: Tau² = 0.06; Chi² = 22.17, df = 11 (P = 0.02); I² = 50%								
Test for overall effect: Z = 5.33 (P < 0.00001)								
**7.1.3 Dose >50 mg**								
Lembcke 1990 (1)	0.181	0.499	8	1.115	0.7	8	4.1%	-0.93 [-1.53, -0.34]
Lembcke 1990 (2)	0.084	0.211	8	0.647	0.44	8	7.1%	-0.56 [-0.90, -0.22]
Lembcke 1991a (3)	0.077	0.189	8	0.714	0.58	8	6.0%	-0.64 [-1.06, -0.21]
Lembcke 1991a (6)	0.271	0.264	8	0.73	0.335	8	7.7%	-0.46 [-0.75, -0.16]
Renard 1991	4.904	0.677	16	6.556	1.247	16	3.4%	-1.65 [-2.35, -0.96]
**Subtotal (95% CI)**			**48**			**48**	**28.3%**	**-0.75 [-1.07, -0.43]**
Heterogeneity: Tau² = 0.08; Chi² = 10.71, df = 4 (P = 0.03); I² = 63%								
Test for overall effect: Z = 4.62 (P < 0.00001)								
**Total (95% CI)**			**219**			**219**	**100.0%**	**-0.57 [-0.72, -0.41]**
Heterogeneity: Tau² = 0.06; Chi² = 38.92, df = 19 (P = 0.005); I² = 51%								
Test for overall effect: Z = 7.21 (P < 0.00001)								
Test for subgroup differences: Chi² = 6.23, df = 2 (P = 0.04), I² = 67.9%								

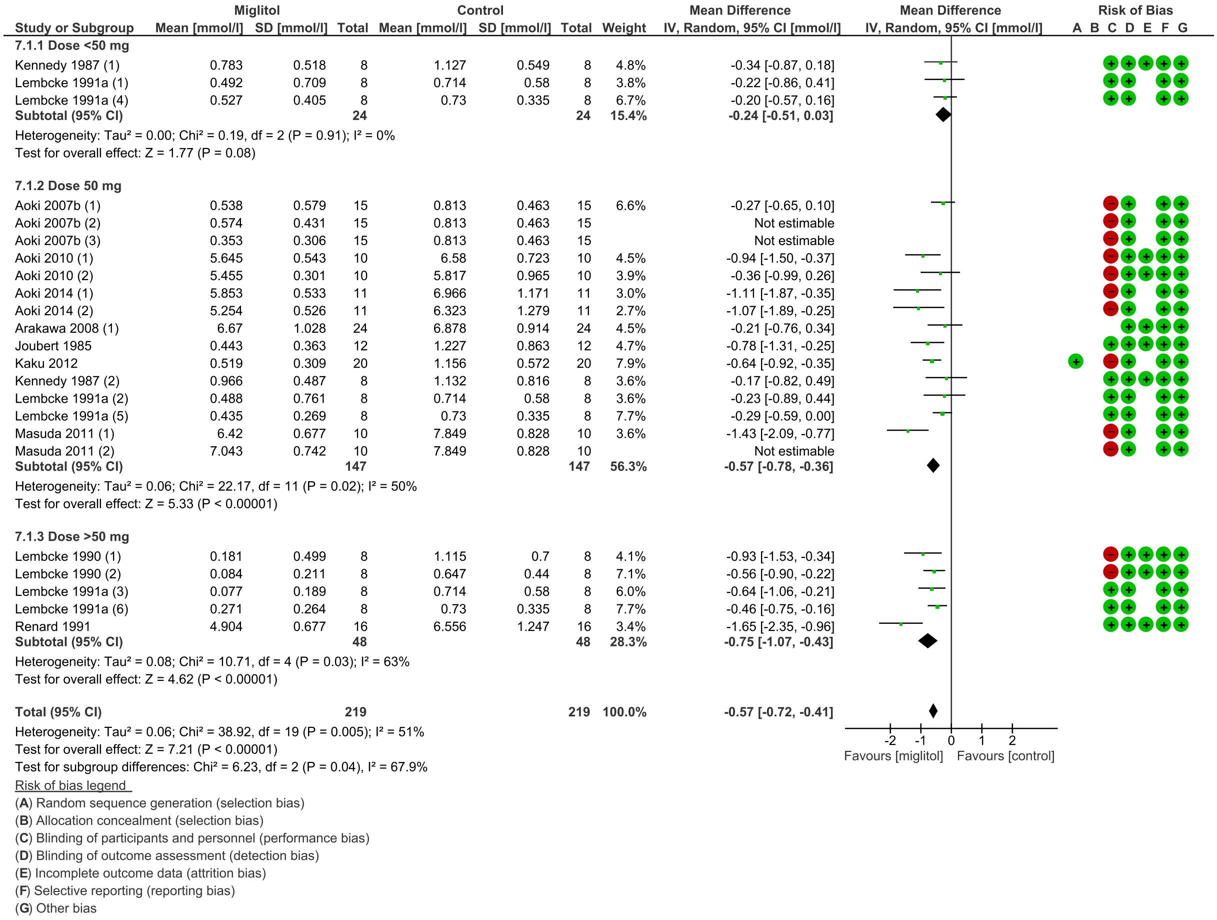

Risk of bias legend
(**A**) Random sequence generation (selection bias)
(**B**) Allocation concealment (selection bias)
(**C**) Blinding of participants and personnel (performance bias)
(**D**) Blinding of outcome assessment (detection bias)
(**E**) Incomplete outcome data (attrition bias)
(**F**) Selective reporting (reporting bias)
(**G**) Other bias

Supplementary Figure 1e. Forest plot of miglitol studies on mean postprandial glucose response levels in individuals without diabetes.

Study or Subgroup	Miglitol Mean [mmol/l]	Miglitol SD [mmol/l]	Miglitol Total	Control Mean [mmol/l]	Control SD [mmol/l]	Control Total	Weight	Mean Difference IV, Random, 95% CI [mmol/l]
**8.1.1 Dose 50 mg**								
Aoki 2007a (1)	11.082	1.243	13	13.249	1.775	13	4.6%	-2.17 [-3.34, -0.99]
Aoki 2007a (2)	11.476	1.243	13	13.249	1.775	13		Not estimable
Aoki 2007a (3)	11.082	1.599	13	13.249	1.775	13		Not estimable
Aoki 2012	9.213	2.396	12	10.173	3.358	12	1.3%	-0.96 [-3.29, 1.37]
Heinz 1989 (2)	1.925	0.196	4	4.192	0.9	4	7.0%	-2.27 [-3.17, -1.36]
Hiki 2010 (1)	8.64	1.645	11	10.225	1.745	11	3.3%	-1.58 [-3.00, -0.17]
Joubert 1986	2.025	3.295	10	3.95	2.816	10	1.0%	-1.93 [-4.61, 0.76]
Katsilambros 1986	1.481	1.07	12	2.429	1.311	12	6.4%	-0.95 [-1.91, 0.01]
Scott 1988 (1)	1.372	0.953	20	2.601	1.009	20	11.7%	-1.23 [-1.84, -0.62]
Shibata 2016 (1)	2.31	2.38	13	4.74	2.66	13	1.9%	-2.43 [-4.37, -0.49]
Shibata 2016 (2)	1.98	2.81	13	4.74	2.66	13	0.0%	-2.76 [-4.86, -0.66]
Ueno 2015 (1)	3.099	0.426	11	4.388	0.513	11	17.5%	-1.29 [-1.68, -0.89]
Wing 1990	4.516	2.5	11	7.318	2.876	11	1.4%	-2.80 [-5.05, -0.55]
**Subtotal (95% CI)**			**117**			**117**	**56.2%**	**-1.46 [-1.76, -1.17]**
Heterogeneity: Tau² = 0.01; Chi² = 9.46, df = 9 (P = 0.40); I² = 5%								
Test for overall effect: Z = 9.72 (P < 0.00001)								
**8.1.2 Dose >50 mg**								
Dimitriadis 1991 (1)	0.689	1.14	12	3.034	2.275	12	3.2%	-2.34 [-3.78, -0.91]
Dimitriadis 1991 (2)	0.084	0.996	12	1.855	1.376	12	6.3%	-1.77 [-2.73, -0.81]
Heinz 1989 (3)	2.238	0.688	4	4.758	1.838	4	1.9%	-2.52 [-4.44, -0.60]
Heinz 1989 (4)	1.575	0.442	4	4.192	0.9	4	6.1%	-2.62 [-3.60, -1.63]
Heinz 1989 (5)	2.379	0.3	4	4.758	1.838	4		Not estimable
Kingma 1992 (1)	1.126	1.705	18	2.467	1.578	18	5.3%	-1.34 [-2.41, -0.27]
Kingma 1992 (2)	1.436	2.021	18	3.719	1.841	18	4.1%	-2.28 [-3.55, -1.02]
Kingma 1992 (3)	1.82	1.505	18	4.562	2.076	18	4.5%	-2.74 [-3.93, -1.56]
Scott 1988 (2)	0.74	0.869	20	2.601	1.009	20	12.3%	-1.86 [-2.44, -1.28]
Scott 1988 (3)	0.96	0.785	20	2.601	1.009	20		Not estimable
Scott 1988 (4)	0.229	0.869	20	2.601	1.009	20		Not estimable
**Subtotal (95% CI)**			**106**			**106**	**43.8%**	**-2.05 [-2.41, -1.70]**
Heterogeneity: Tau² = 0.00; Chi² = 5.51, df = 7 (P = 0.60); I² = 0%								
Test for overall effect: Z = 11.39 (P < 0.00001)								
**Total (95% CI)**			**223**			**223**	**100.0%**	**-1.77 [-2.05, -1.50]**

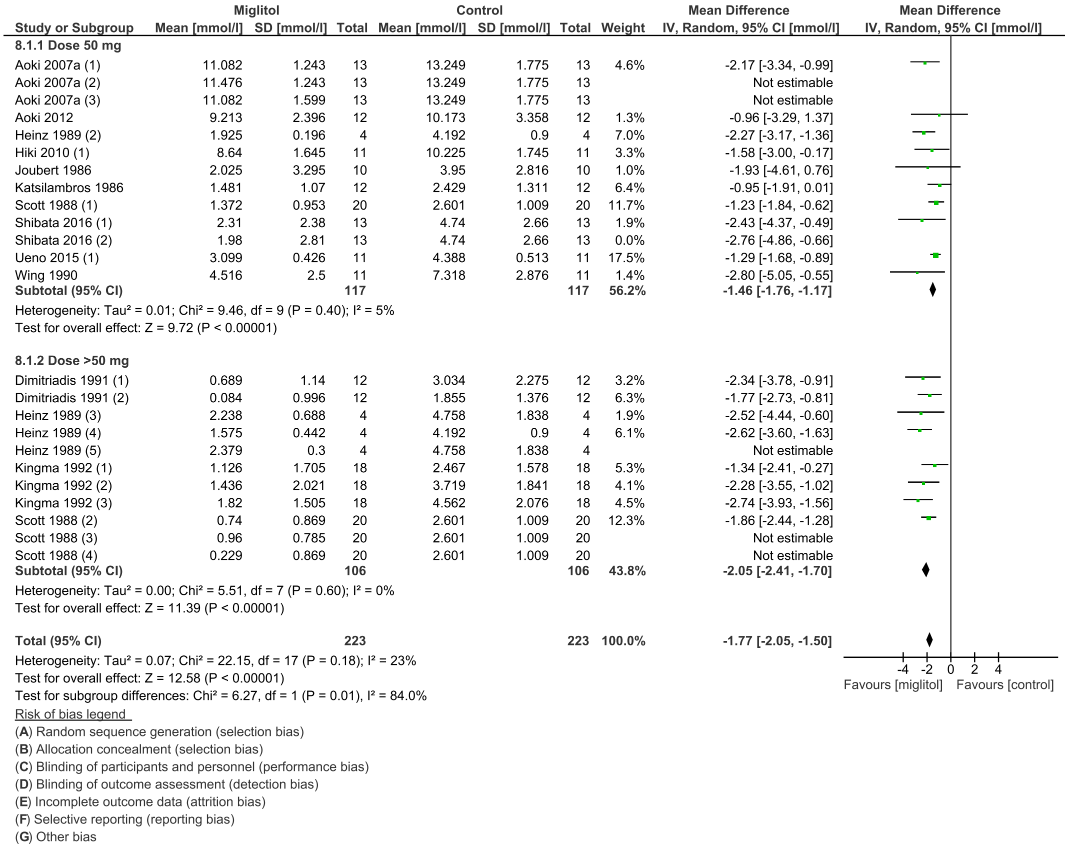

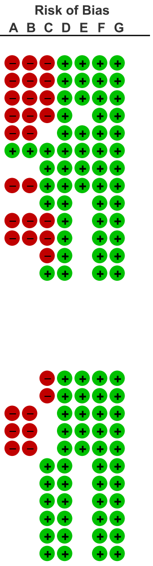

Heterogeneity: Tau² = 0.07; Chi² = 22.15, df = 17 (P = 0.18); I² = 23%
Test for overall effect: Z = 12.58 (P < 0.00001)
Test for subgroup differences: Chi² = 6.27, df = 1 (P = 0.01), I² = 84.0%

Risk of bias legend
(**A**) Random sequence generation (selection bias)
(**B**) Allocation concealment (selection bias)
(**C**) Blinding of participants and personnel (performance bias)
(**D**) Blinding of outcome assessment (detection bias)
(**E**) Incomplete outcome data (attrition bias)
(**F**) Selective reporting (reporting bias)
(**G**) Other bias

Supplementary Figure 1f. Forest plot of miglitol studies on mean postprandial glucose response levels in individuals with diabetes.

Study or Subgroup	Miglitol Mean [pmol/l]	Miglitol SD [pmol/l]	Miglitol Total	Control Mean [pmol/l]	Control SD [pmol/l]	Control Total	Weight	Mean Difference IV, Random, 95% CI [pmol/l]
**7.2.1 Dose <50 mg**								
Kennedy 1987 (1)	147	14.14	8	196.8	93.34	8	4.8%	-49.80 [-115.22, 15.62]
Lembcke 1991a (1)	83.33	47.14	8	70	66	8	5.5%	13.33 [-42.87, 69.53]
Lembcke 1991a (4)	30	18.86	8	66.67	37.71	8	7.9%	-36.67 [-65.89, -7.45]
**Subtotal (95% CI)**			**24**			**24**	**18.2%**	**-26.84 [-58.93, 5.26]**
Heterogeneity: Tau² = 259.40; Chi² = 2.83, df = 2 (P = 0.24); I² = 29%								
Test for overall effect: Z = 1.64 (P = 0.10)								
**7.2.2 Dose 50 mg**								
Aoki 2007b (1)	194.97	85.59	15	332.67	167.31	15	3.1%	-137.70 [-232.80, -42.60]
Aoki 2007b (2)	164.83	70.1	15	332.67	167.31	15		Not estimable
Aoki 2007b (3)	120.6	54.48	15	332.67	167.31	15		Not estimable
Aoki 2010 (1)	209.2	140.62	10	397.2	269.95	10	1.0%	-188.00 [-376.65, 0.65]
Aoki 2010 (2)	194.27	97.82	10	351.43	168.23	10	2.2%	-157.16 [-277.77, -36.55]
Aoki 2014 (1)	193.04	189.93	11	474.44	377.38	11	0.6%	-281.40 [-531.06, -31.74]
Aoki 2014 (2)	137.07	88.67	11	357.59	230.69	11	1.6%	-220.52 [-366.57, -74.47]
Arakawa 2008 (1)	166.38	75.85	24	222.43	86.2	24	6.4%	-56.05 [-101.99, -10.11]
Joubert 1985	4.63	3.03	12	17.07	8.3	12	9.5%	-12.44 [-17.44, -7.44]
Kaku 2012	85.07	54.04	20	165.17	81.63	20	6.7%	-80.10 [-123.00, -37.20]
Kennedy 1987 (2)	120.6	14.71	8	169.8	83.16	8	5.3%	-49.20 [-107.72, 9.32]
Lembcke 1991a (2)	46.67	37.71	8	70	66	8	5.8%	-23.33 [-76.00, 29.34]
Lembcke 1991a (5)	26.67	18.86	8	66.67	37.71	8	7.9%	-40.00 [-69.22, -10.78]
**Subtotal (95% CI)**			**137**			**137**	**50.1%**	**-67.61 [-99.11, -36.10]**
Heterogeneity: Tau² = 1486.10; Chi² = 43.96, df = 10 (P < 0.00001); I² = 77%								
Test for overall effect: Z = 4.21 (P < 0.0001)								
**7.2.3 Dose >50 mg**								
Lembcke 1990 (1)	17.87	28.96	8	112.67	74.83	8	5.5%	-94.80 [-150.40, -39.20]
Lembcke 1990 (2)	11.16	16.67	8	69.59	41.43	8	7.8%	-58.43 [-89.38, -27.48]
Lembcke 1991a (3)	3.33	9.43	8	70	66	8	6.4%	-66.67 [-112.87, -20.47]
Lembcke 1991a (6)	10	18.86	8	66.67	37.71	8	7.9%	-56.67 [-85.89, -27.45]
Renard 1991	147.05	82.6	16	275.45	131.4	16	4.1%	-128.40 [-204.45, -52.35]
**Subtotal (95% CI)**			**48**			**48**	**31.7%**	**-67.04 [-85.55, -48.52]**
Heterogeneity: Tau² = 26.80; Chi² = 4.24, df = 4 (P = 0.37); I² = 6%								
Test for overall effect: Z = 7.10 (P < 0.00001)								
**Total (95% CI)**			**209**			**209**	**100.0%**	**-60.44 [-80.71, -40.17]**
Heterogeneity: Tau² = 1123.94; Chi² = 80.84, df = 18 (P < 0.00001); I² = 78%								
Test for overall effect: Z = 5.84 (P < 0.00001)								
Test for subgroup differences: Chi² = 4.87, df = 2 (P = 0.09), I² = 58.9%								

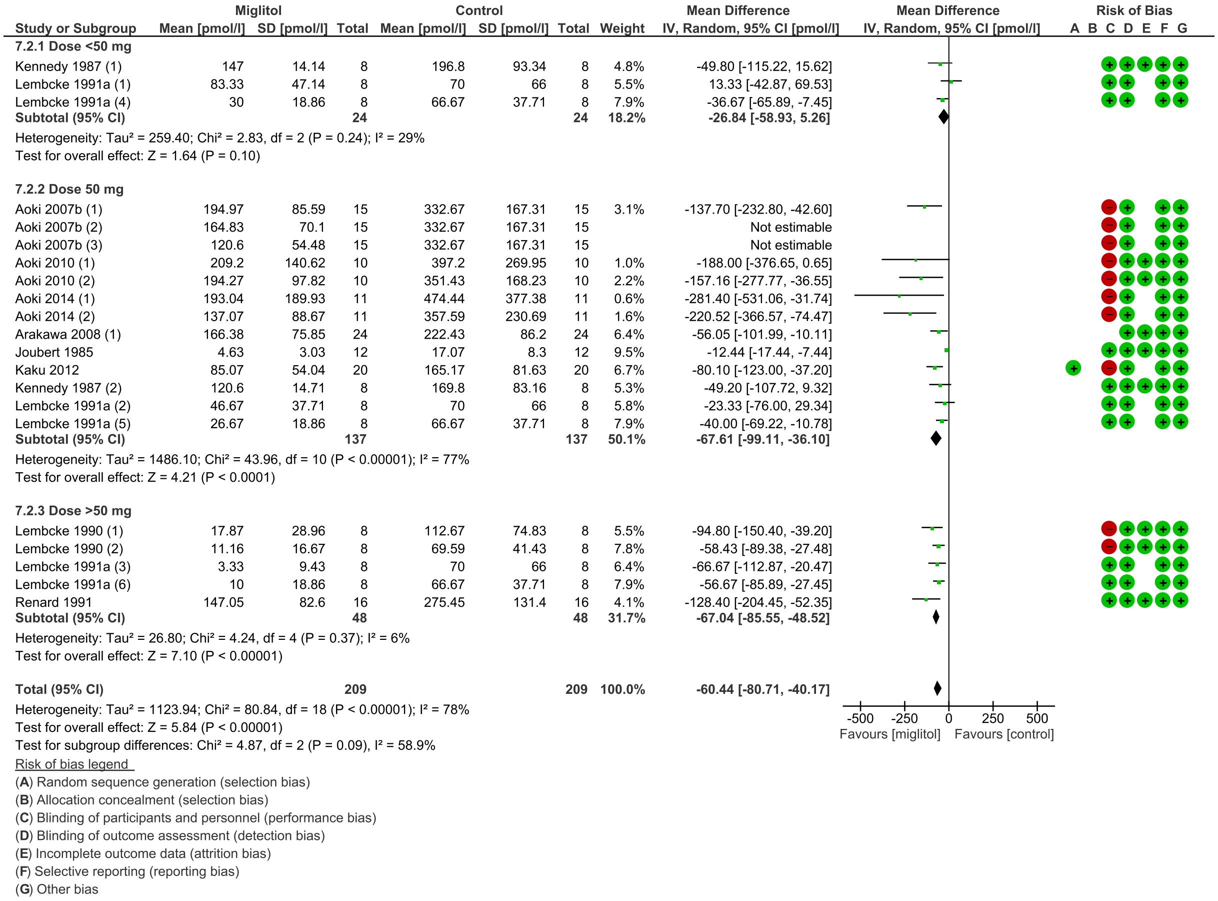

Risk of bias legend
(**A**) Random sequence generation (selection bias)
(**B**) Allocation concealment (selection bias)
(**C**) Blinding of participants and personnel (performance bias)
(**D**) Blinding of outcome assessment (detection bias)
(**E**) Incomplete outcome data (attrition bias)
(**F**) Selective reporting (reporting bias)
(**G**) Other bias

Supplementary Figure 1g. Forest plot of miglitol studies on mean postprandial insulin response levels in individuals without diabetes.

Study or Subgroup	Miglitol Mean [pmol/l]	Miglitol SD [pmol/l]	Miglitol Total	Control Mean [pmol/l]	Control SD [pmol/l]	Control Total	Weight	Mean Difference IV, Random, 95% CI [pmol/l]
**8.2.1 Dose 50 mg**								
Aoki 2007a (1)	121	88.34	13	160.6	150.59	13	0.8%	-39.60 [-134.51, 55.31]
Aoki 2007a (2)	149.07	80.52	13	160.6	150.59	13		Not estimable
Aoki 2007a (3)	128.9	77.88	13	160.6	150.59	13		Not estimable
Aoki 2012	234.99	405.63	12	281.23	571.46	12	0.0%	-46.24 [-442.74, 350.26]
Heinz 1989 (2)	26.25	12.75	4	26.25	15	4	11.1%	0.00 [-19.29, 19.29]
Hiki 2010 (1)	147.25	68.45	11	251.6	95.25	11	1.5%	-104.35 [-173.67, -35.03]
Joubert 1986	50.6	53.37	10	77.9	71.18	10	2.3%	-27.30 [-82.44, 27.84]
Scott 1988 (1)	92.7	52.21	20	96.64	57.06	20	5.2%	-3.94 [-37.84, 29.96]
Shibata 2016 (1)	72.27	56.32	13	117.1	83.71	13	2.3%	-44.83 [-99.67, 10.01]
Shibata 2016 (2)	64.23	50.23	13	117.1	83.71	13	0.0%	-52.87 [-105.94, 0.20]
Ueno 2015 (1)	117.89	27.87	11	167.25	42.97	11	6.2%	-49.36 [-79.63, -19.09]
**Subtotal (95% CI)**			**94**			**94**	**29.5%**	**-30.22 [-54.65, -5.79]**
Heterogeneity: Tau² = 546.37; Chi² = 15.31, df = 7 (P = 0.03); I² = 54%								
Test for overall effect: Z = 2.42 (P = 0.02)								
**8.2.2 Dose >50 mg**								
Heinz 1989 (3)	9.35	7.5	4	13.35	9.9	4	16.3%	-4.00 [-16.17, 8.17]
Heinz 1989 (4)	23.7	15	4	26.25	15	4	10.2%	-2.55 [-23.34, 18.24]
Heinz 1989 (5)	11.78	7.95	4	13.35	9.9	4	16.0%	-1.57 [-14.01, 10.87]
Kingma 1992 (1)	71.33	50.38	18	70.55	32	18	7.1%	0.78 [-26.79, 28.35]
Kingma 1992 (2)	57.08	39.42	18	74.95	34.34	18	8.5%	-17.87 [-42.02, 6.28]
Kingma 1992 (3)	70.95	47.9	18	78.06	35.79	18	7.1%	-7.11 [-34.73, 20.51]
Scott 1988 (2)	75.96	50.99	20	96.64	57.06	20	5.3%	-20.68 [-54.22, 12.86]
Scott 1988 (3)	64.31	52.21	20	96.64	57.06	20		Not estimable
Scott 1988 (4)	63	52.21	20	96.64	57.06	20		Not estimable
**Subtotal (95% CI)**			**86**			**86**	**70.5%**	**-4.84 [-11.78, 2.11]**
Heterogeneity: Tau² = 0.00; Chi² = 2.49, df = 6 (P = 0.87); I² = 0%								
Test for overall effect: Z = 1.37 (P = 0.17)								
**Total (95% CI)**			**180**			**180**	**100.0%**	**-11.09 [-19.88, -2.30]**
Heterogeneity: Tau² = 84.99; Chi² = 21.26, df = 14 (P = 0.10); I² = 34%								
Test for overall effect: Z = 2.47 (P = 0.01)								
Test for subgroup differences: Chi² = 3.84, df = 1 (P = 0.05), I² = 73.9%								

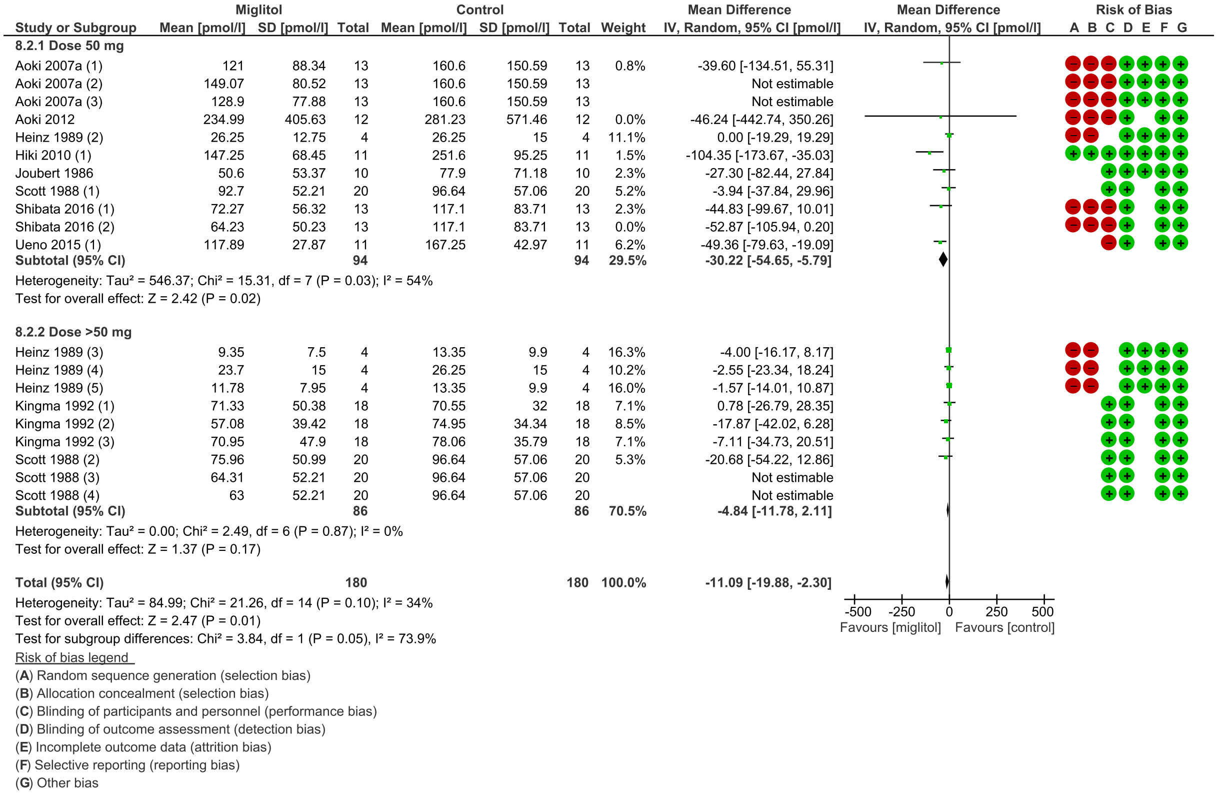

Risk of bias legend
(**A**) Random sequence generation (selection bias)
(**B**) Allocation concealment (selection bias)
(**C**) Blinding of participants and personnel (performance bias)
(**D**) Blinding of outcome assessment (detection bias)
(**E**) Incomplete outcome data (attrition bias)
(**F**) Selective reporting (reporting bias)
(**G**) Other bias

Supplementary Figure 1h. Forest plot of miglitol studies on mean postprandial insulin response levels in individuals with diabetes.

	Voglibose			Control				Mean Difference	Mean Difference	Risk of Bias						
**Study or Subgroup**	**Mean [mmol/l]**	**SD [mmol/l]**	**Total**	**Mean [mmol/l]**	**SD [mmol/l]**	**Total**	**Weight**	**IV, Random, 95% CI [mmol/l]**	**IV, Random, 95% CI [mmol/l]**	**A**	**B**	**C**	**D**	**E**	**F**	**G**
Aoki 2009 (1)	6.142	0.934	16	6.608	1.133	16	19.9%	-0.47 [-1.19, 0.25]				−	+		+	+
Hiki 2010 (2)	10.03	1.765	11	10.225	1.745	11	4.8%	-0.20 [-1.66, 1.27]		+	+	+	+	+	+	+
Kagayama 1997 (4)	5.578	0.645	20	5.644	0.757	20		Not estimable				+	+	+	+	+
Kageyama 1997 (3)	5.547	0.869	20	5.644	0.757	20	40.4%	-0.10 [-0.60, 0.41]				+	+	+	+	+
Kageyama 2000 (2)	6.166	0.613	12	6.666	0.74	12	34.9%	-0.50 [-1.04, 0.04]				+	+	+	+	+
**Total (95% CI)**			**59**			**59**	**100.0%**	**-0.32 [-0.64, 0.01]**								

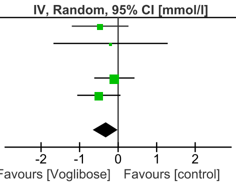

Heterogeneity: Tau² = 0.00; Chi² = 1.36, df = 3 (P = 0.72); I² = 0%

Test for overall effect: Z = 1.93 (P = 0.05)

Risk of bias legend

(**A**) Random sequence generation (selection bias)
(**B**) Allocation concealment (selection bias)
(**C**) Blinding of participants and personnel (performance bias)
(**D**) Blinding of outcome assessment (detection bias)
(**E**) Incomplete outcome data (attrition bias)
(**F**) Selective reporting (reporting bias)
(**G**) Other bias

Supplementary Figure 1i. Forest plot of voglibose studies on mean postprandial glucose response levels.

	Voglibose			Control				Mean Difference	Mean Difference	Risk of Bias						
**Study or Subgroup**	**Mean [pmol/l]**	**SD [pmol/l]**	**Total**	**Mean [pmol/l]**	**SD [pmol/l]**	**Total**	**Weight**	**IV, Random, 95% CI [pmol/l]**	**IV, Random, 95% CI [pmol/l]**	**A**	**B**	**C**	**D**	**E**	**F**	**G**
Aoki 2009 (1)	226.9	116.93	16	267.83	144.93	16	25.5%	-40.93 [-132.18, 50.32]				−	+		+	+
Aoki 2009 (2)	235.1	112.27	16	267.83	144.93	16		Not estimable				−	+		+	+
Goke 1995 (1)	240.97	178.97	12	310.93	233.33	12	7.7%	-69.96 [-236.34, 96.42]				+	+		+	+
Goke 1995 (2)	206.97	122.97	12	310.93	233.33	12		Not estimable				+	+		+	+
Goke 1995 (3)	139.97	42	12	310.93	233.33	12		Not estimable				+	+		+	+
Goke 1995 (4)	105.97	35	12	310.93	233.33	12		Not estimable				+	+		+	+
Hiki 2010 (2)	244.15	102.4	11	251.6	95.25	11	31.1%	-7.45 [-90.10, 75.20]		+	+	+	+	+	+	+
Kagayama 1997 (4)	277.8	167.71	20	321.3	171.73	20		Not estimable				+	+	+	+	+
Kageyama 1997 (3)	289.2	159.66	20	321.3	171.73	20	20.1%	-32.10 [-134.87, 70.67]				+	+	+	+	+
Kageyama 2000 (2)	366.9	148.71	12	406.8	143.41	12	15.6%	-39.90 [-156.79, 76.99]				+	+	+	+	+
**Total (95% CI)**			**71**			**71**	**100.0%**	**-30.80 [-76.90, 15.30]**								

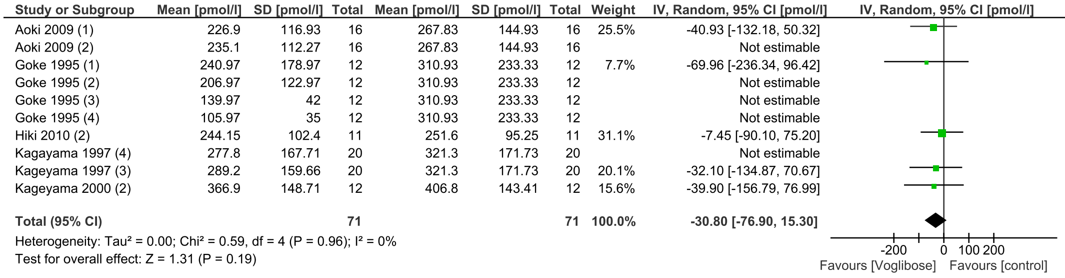

Heterogeneity: Tau² = 0.00; Chi² = 0.59, df = 4 (P = 0.96); I² = 0%

Test for overall effect: Z = 1.31 (P = 0.19)

Risk of bias legend

(**A**) Random sequence generation (selection bias)
(**B**) Allocation concealment (selection bias)
(**C**) Blinding of participants and personnel (performance bias)
(**D**) Blinding of outcome assessment (detection bias)
(**E**) Incomplete outcome data (attrition bias)
(**F**) Selective reporting (reporting bias)
(**G**) Other bias

Supplementary Figure 1j. Forest plot of voglibose studies on mean postprandial insulin response levels.

Study or Subgroup	Emiglitate Mean [mmol/l]	Emiglitate SD [mmol/l]	Emiglitate Total	Control Mean [mmol/l]	Control SD [mmol/l]	Control Total	Weight	Mean Difference IV, Random, 95% CI [mmol/l]	A	B	C	D	E	F	G
Kennedy 1987 (3)	0.622	0.22	7	0.771	0.264	7	32.6%	-0.15 [-0.40, 0.11]			+	+	+	+	+
Kennedy 1987 (4)	0.572	0.235	8	0.788	0.283	8	32.5%	-0.22 [-0.47, 0.04]			+	+	+	+	+
Lembcke 1991b (1)	0.083	0.182	8	0.723	0.476	8	24.4%	-0.64 [-0.99, -0.29]			+	+	+	+	+
Lembcke 1991b (2)	0.001	0.189	8	0.723	0.476	8	0.0%	-0.72 [-1.08, -0.37]			+	+	+	+	+
Lembcke 1991b (3)	0.095	0.237	8	0.723	0.476	8	0.0%	-0.63 [-1.00, -0.26]			+	+	+	+	+
Lembcke 1991b (4)	0.789	0.534	8	0.733	0.795	8	10.5%	0.06 [-0.61, 0.72]			+	+	+	+	+
Lembcke 1991b (5)	0.706	0.365	8	0.733	0.795	8	0.0%	-0.03 [-0.63, 0.58]			+	+	+	+	+
Lembcke 1991b (6)	0.069	0.317	8	0.733	0.795	8	0.0%	-0.66 [-1.26, -0.07]			+	+	+	+	+
**Total (95% CI)**			**31**			**31**	**100.0%**	**-0.27 [-0.51, -0.03]**							

Heterogeneity: Tau² = 0.03; Chi² = 6.16, df = 3 (P = 0.10); I² = 51%
Test for overall effect: Z = 2.19 (P = 0.03)

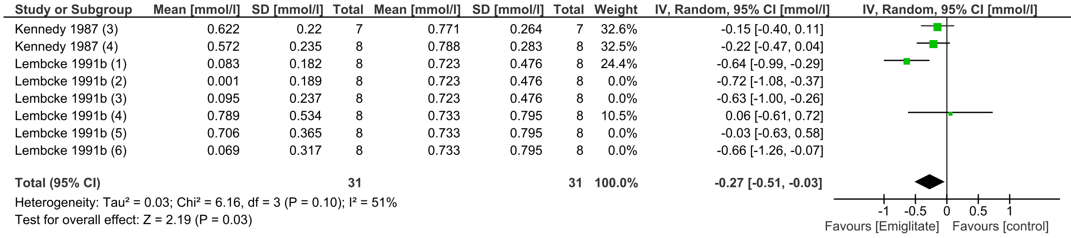

Risk of bias legend
(**A**) Random sequence generation (selection bias)
(**B**) Allocation concealment (selection bias)
(**C**) Blinding of participants and personnel (performance bias)
(**D**) Blinding of outcome assessment (detection bias)
(**E**) Incomplete outcome data (attrition bias)
(**F**) Selective reporting (reporting bias)
(**G**) Other bias

Supplementary Figure 1k. Forest plot of emiglitate studies on mean postprandial glucose response levels.

Study or Subgroup	Emiglitate Mean [pmol/l]	Emiglitate SD [pmol/l]	Emiglitate Total	Control Mean [pmol/l]	Control SD [pmol/l]	Control Total	Weight	Mean Difference IV, Random, 95% CI [pmol/l]	A	B	C	D	E	F	G
Kennedy 1987 (3)	128.4	15.35	7	163.8	68.26	7	17.7%	-35.40 [-87.23, 16.43]			+	+	+	+	+
Kennedy 1987 (4)	168	24.32	8	235.8	112.01	8	7.5%	-67.80 [-147.23, 11.63]			+	+	+	+	+
Lembcke 1991b (1)	13.33	18.86	8	63.33	37.71	8	55.6%	-50.00 [-79.22, -20.78]			+	+	+	+	+
Lembcke 1991b (2)	6.67	9.43	8	63.33	37.71	8	0.0%	-56.66 [-83.60, -29.72]			+	+	+	+	+
Lembcke 1991b (3)	3.33	9.43	8	63.33	37.71	8	0.0%	-60.00 [-86.94, -33.06]			+	+	+	+	+
Lembcke 1991b (4)	50	28.28	8	76.67	66	8	19.2%	-26.67 [-76.43, 23.09]			+	+	+	+	+
Lembcke 1991b (5)	30	18.86	8	76.67	66	8	0.0%	-46.67 [-94.24, 0.90]			+	+	+	+	+
Lembcke 1991b (6)	-3.33	18.86	8	76.67	66	8	0.0%	-80.00 [-127.57, -32.43]			+	+	+	+	+
**Total (95% CI)**			**31**			**31**	**100.0%**	**-44.28 [-66.07, -22.49]**							

Heterogeneity: Tau² = 0.00; Chi² = 1.08, df = 3 (P = 0.78); I² = 0%
Test for overall effect: Z = 3.98 (P < 0.0001)

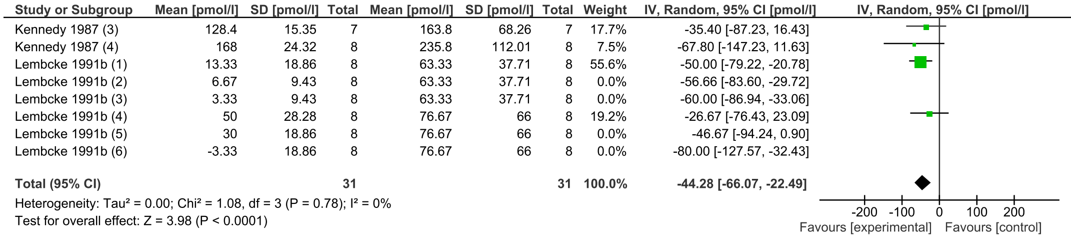

Risk of bias legend
(**A**) Random sequence generation (selection bias)
(**B**) Allocation concealment (selection bias)
(**C**) Blinding of participants and personnel (performance bias)
(**D**) Blinding of outcome assessment (detection bias)
(**E**) Incomplete outcome data (attrition bias)
(**F**) Selective reporting (reporting bias)
(**G**) Other bias

Supplementary Figure 1l. Forest plot of emiglitate studies on mean postprandial insulin response levels.

Supplementary Table 1a: Characteristics of Study Comparisons for Acarbose

Author (comparison)(reference)	Ref	Study Design	Randomised	N	Health status	Dose	Timing (0-before; 1-with)	Meal test	Carbs (g)	Duration (min)	PPG	PPI
Arakawa 2008 (2)	(1)	cross-over	yes	24	no DM	100 mg	0	mixed meal	56.5	240	yes	yes
Aziz 2013 (1)	(2)	cross-over	unk	13	no DM	50 mg	0	sucrose	70	180	yes	no
Aziz 2013 (2)	(2)	cross-over	unk	13	no DM	100 mg	0	sucrose	70	180	yes	no
Caspary 1978	(3)	cross-over	yes	7	no DM	200 mg	1	sucrose	100	180	yes	no
Ceriello 1996	(4)	cross-over	yes	17	DM	100 mg	0	mixed meal	45.6	240	yes	yes
Chen 2016	(5)	cross-over	no	12	DM	100 mg	1	carb meal	68.5	120	yes	yes
Debeljak 2016	(6)	cross-over	yes	31	no DM	50 mg	1	carb meal	50	120	yes	no
Deleon 2002 (1)	(7)	cross-over	partially	10	DM	25 mg	1	mixed meal	61	120	yes	yes
Deleon 2002 (2)	(7)	cross-over	partially	10	DM	50 mg	1	mixed meal	61	120	yes	yes
Deleon 2002 (3)	(7)	cross-over	partially	10	DM	100 mg	1	mixed meal	61	120	yes	yes
Dimitriadis 1982	(8)	cross-over	unk	6	No DM	100 mg	0	mixed meal	79	240	yes	yes
Enc 2001 (1)	(9)	cross-over	yes	14	No DM	100 mg	1	mixed meal	72.6	180	yes	yes
Enc 2001 (2)	(9)	cross-over	yes	12	No DM	100 mg	1	sucrose	50	120	yes	yes
Frank 1998 (1)	(10)	cross-over	yes	20	DM	100 mg	1	mixed meal	60	210	yes	no
Frank 1998 (2)	(10)	cross-over	yes	20	DM	100 mg	1	mixed meal	40	120	yes	no
Frati Munari 1998 (1)	(11)	cross-over	yes	12	DM	200 mg	0	carb meal	50	180	yes	yes
Frati Munari 1998 (2)	(11)	cross-over	yes	10	no DM	200 mg	0	carb meal	50	180	yes	yes
Gentilcore 2005	(12)	cross-over	yes	8	no DM	100 mg	1	sucrose	100	210	yes	yes
Hermanns 2004	(13)	cross-over	yes	30	DM	100 mg	1	mixed meal	53.5	240	yes	no
Hillebrand 1979a (1)	(14)	cross-over	yes	12	no DM	75 mg	0	mixed meal	90	180	yes	yes
Hillebrand 1979a (2)	(14)	cross-over	yes	12	no DM	150 mg	0	mixed meal	90	180	yes	yes
Hillebrand 1979a (3)	(14)	cross-over	yes	12	no DM	300 mg	0	mixed meal	90	180	yes	yes
Hillebrand 1979b (1)	(15)	cross-over	yes	24	no DM	50 mg	0	mixed meal	111	180	yes	yes
Hillebrand 1979b (2)	(15)	cross-over	yes	24	no DM	100 mg	0	mixed meal	111	180	yes	yes

Hillebrand 1979b (3)	(14)	cross-over	yes	24	no DM	200 mg	0	mixed meal	111	180	yes	yes
Hopfner 1997 (1)	(16)	cross-over	yes	24	no DM	100 mg	0	sucrose	75	240	yes	yes
Hopfner 1997 (2)	(16)	cross-over	yes	24	no DM	100 mg	0	sucrose	75	240	yes	yes
Hucking 2005	(17)	cross-over	yes	10	DM	100 mg	1	mixed meal	72.4	360	yes	yes
Jenkins 1979 (1)	(18)	cross-over	yes	8	no DM	50 mg	0	mixed meal	85	120	yes	yes
Jenkins 1979 (2)	(18)	cross-over	yes	8	no DM	50 mg	0	mixed meal	85	120	yes	yes
Jenney 1993	(19)	cross-over	unk	5	DM	25 mg	1	mixed meal	75	120	yes	yes
Kado 1998	(20)	cross-over	no	20	DM	100 mg	0	mixed meal	31.8	180	yes	yes
Kageyama 1997 (1)	(21)	cross-over	yes	20	no DM	50 mg	0	mixed meal	82.4	120	yes	yes
Kageyama 1997 (2)	(21)	cross-over	yes	20	no DM	100 mg	0	mixed meal	82.4	120	yes	yes
Kageyama 2000 (1)	(22)	cross-over	yes	12	no DM	100 mg	0	mixed meal	82.4	120	yes	yes
Koytchev 2009 (1)	(23)	cross-over	yes	12	no DM	50 mg	1	sucrose	50	120	yes	no
Koytchev 2009 (2)	(23)	cross-over	yes	12	no DM	200 mg	1	sucrose	50	120	yes	no
Koytchev 2009 (3)	(23)	cross-over	yes	12	no DM	50 mg	1	carb meal	50	120	yes	no
Koytchev 2009 (4)	(23)	cross-over	yes	12	no DM	200 mg	1	carb meal	50	120	yes	no
Kusunoki 2013	(24)	cross-over	no	10	DM	100 mg	0	mixed meal	56.5	180	yes	yes
Lee 2012 (1)	(25)	cross-over	no	5	no DM	200 mg	1	sucrose	100	180	yes	no
Lee 2012 (2)	(25)	cross-over	no	33	no DM	200 mg	1	sucrose	100	180	yes	no
Lee 2012 (3)	(25)	cross-over	no	33	no DM	200 mg	1	sucrose	100	180	yes	no
Lettieri 1998	(26)	cross-over	yes	37	DM	100 mg	1	mixed meal	unk	120	yes	no
O'Dea 1985 (1)	(27)	cross-over	yes	6	no DM	12.5 mg	1	carb meal	71.3	180	yes	yes
O'Dea 1985 (2)	(27)	cross-over	yes	6	no DM	25 mg	1	carb meal	71.3	180	yes	yes
O'Dea 1985 (3)	(27)	cross-over	yes	6	no DM	50 mg	1	carb meal	71.3	180	yes	yes
Ogawa 2004	(28)	cross-over	no	40	DM	100 mg	1	mixed meal	41.7	360	yes	yes
Rabasa-Lhoret 2001	(29)	cross-over	yes	7	DM	100 mg	1	mixed meal	75	90	yes	no
Rosak 2002 (1)	(30)	parallel	yes	22	DM	100 mg	1	mixed meal	53.5	240	yes	yes
Rosak 2002 (2)	(30)	parallel	yes	21	DM	100 mg	1	standard meal	53.5	240	yes	yes
Seifarth 1998	(31)	cross-over	yes	11	DM	100 mg	1	sucrose	100	360	yes	yes
Shibao 2007	(32)	cross-over	yes	13	no DM	100 mg	0	mixed meal	42.3	90	yes	yes

Shimabukuro 2006	(33)	cross-over	yes	14	DM	100 mg	0	mixed meal	57.8	240	yes	yes
Tamasawa 2015	(34)	cross-over	no	16	DM	100 mg	0	mixed meal	Unk	300	yes	no
Taylor 1982 (1)	(35)	cross-over	yes	4	no DM	50 mg	1	Sucrose/starch	55%E	120	yes	no
Taylor 1982 (2)	(35)	cross-over	yes	4	no DM	50 mg	1	carb meal	55%E	120	yes	no
Taylor 1982 (3)	(35)	cross-over	yes	4	no DM	50 mg	1	carb meal	55%E	120	yes	no
Ueno 2015 (2)	(36)	cross-over	yes	11	DM	100 mg	0	mixed meal	71.4	180	yes	yes
Uttenthal 1986	(37)	cross-over	yes	17	DM	100 mg	1	mixed meal	87	240	yes	no
Uttenthal 1987 (1)	(38)	cross-over	yes	5	no DM	50 mg	1	mixed meal	87	240	no	yes
Uttenthal 1987 (2)	(38)	cross-over	yes	5	no DM	100 mg	1	mixed meal	87	240	no	yes
Uttenthal 1987 (3)	(38)	cross-over	yes	5	no DM	200 mg	1	mixed meal	87	240	no	yes
Uttenthal 1987 (4)	(38)	cross-over	yes	5	no DM	400 mg	1	mixed meal	87	240	no	yes
Wachters-H. 2007	(39)	cross-over	unk	7	no DM	12.5 mg	1	carb meal	45.4	120	yes	yes
Walton 1979	(40)	cross-over	no	7	DM	100 mg	1	standard meal	unk	120	yes	no
Yuhong 2014	(41)	cross-over	yes	18	no DM	50 mg	0	Mixed meal	104	180	yes	yes
Zulkarnain 2009	(42)	cross-over	no	12	no DM	50 mg	0	carb meal	80	180	yes	no

Supplementary Table 1b: Characteristics of Study Comparisons for Miglitol

Author	ref	Study Design (cross-over or parallel)	Randomised	N	Health status	Dose	Timing (0-before; 1-with; 2-after)	Meal test	Carbs (g)	Duration (min)	PPG	PPI
Aoki 2007a (1)	(43)	cross-over	unk	13	DM	50 mg	0	mixed meal	Unk	180	yes	yes
Aoki 2007a (2)	(43)	cross-over	unk	13	DM	50 mg	2	mixed meal	Unk	180	yes	yes
Aoki 2007a (3)	(43)	cross-over	unk	13	DM	50 mg	2	mixed meal	Unk	180	yes	yes
Aoki 2007b (1)	(44)	cross-over	yes	15	no DM	50 mg	0	mixed meal	84.6	180	yes	yes
Aoki 2007b (2)	(44)	cross-over	yes	15	no DM	50 mg	2	mixed meal	84.6	180	yes	yes

Aoki 2007b (3)	(44)	cross-over	yes	15	no DM	50 mg		mixed meal	84.6	180	yes	yes
Aoki 2010 (1)	(45)	cross-over	yes	10	no DM	50 mg	0	mixed meal	121.5	180	yes	yes
Aoki 2010 (2)	(45)	cross-over	yes	10	no DM	50 mg	0	mixed meal	121.5	180	yes	yes
Aoki 2012	(46)	cross-over	no	12	DM	50 mg	0	mixed meal	121.5	240	yes	yes
Aoki 2014 (1)	(47)	cross-over	yes	11	no DM	50 mg	0	mixed meal	125	120	yes	yes
Aoki 2014 (2)	(47)	cross-over	yes	11	no DM	50 mg	0	mixed meal	125	120	yes	yes
Arakawa 2008 (1)	(1)	cross-over	yes	24	no DM	50 mg	0	mixed meal	56.5	240	yes	yes
Dimitriadis 1991 (1)	(48)	cross-over	yes	12	DM	100 mg	0	mixed meal	47.3	300	yes	no
Dimitriadis 1991 (2)	(48)	cross-over	yes	12	DM	100 mg	0	mixed meal	47.3	300	yes	no
Heinz 1989 (1)	(49)	cross-over	no	8	DM	25 mg	unk	mixed meal	50	240	yes	yes
Heinz 1989 (2)	(49)	cross-over	no	4	DM	50 mg	unk	mixed meal	50	240	yes	yes
Heinz 1989 (3)	(49)	cross-over	no	4	DM	75 mg	unk	mixed meal	50	240	yes	yes
Heinz 1989 (4)	(49)	cross-over	no	4	DM	100 mg	unk	mixed meal	50	240	yes	yes
Heinz 1989 (5)	(49)	cross-over	no	4	DM	200 mg	unk	mixed meal	50	240	yes	yes
Hiki 2010 (1)	(50)	cross-over	yes	11	DM	50 mg	0	mixed meal	56.5	120	yes	yes
Joubert 1985	(51)	cross-over	yes	12	no DM	50 mg	1	Carb meal	56	180	yes	yes
Joubert 1986	(52)	cross-over	yes	10	DM	50 mg	1	Carb meal	56	180	yes	yes
Kaku 2012	(53)	cross-over	yes	20	no DM	50 mg	0	mixed meal	75	180	yes	yes
Katsilambros 1986	(54)	cross-over	unk	12	DM	50 mg	1	mixed meal	34.4	180	yes	no
Kennedy 1987 (1)	(55)	cross-over	yes	8	no DM	25 mg	1	mixed meal	101.3	180	yes	yes
Kennedy 1987 (2)	(55)	cross-over	yes	8	no DM	50 mg	1	mixed meal	101.3	180	yes	yes
Kingma 1992 (1)	(56)	parallel	yes	18	DM	100 mg	1	mixed meal	62.7	210	yes	yes
Kingma 1992 (2)	(56)	parallel	yes	18	DM	100 mg	1	mixed meal	62.2	210	yes	yes
Kingma 1992 (3)	(56)	parallel	yes	18	DM	100 mg	1	mixed meal	65.3	210	yes	yes
Lembcke 1990 (1)	(57)	parallel	yes	8	no DM	100 mg	0	sucrose	50	240	yes	yes
Lembcke 1990 (2)	(57)	parallel	yes	8	no DM	100 mg	0	starch	50	240	yes	yes
Lembcke 1991a (1)	(58)	cross-over	yes	8	no DM	25 mg	0	sucrose	50	180	yes	yes
Lembcke 1991a (2)	(58)	cross-over	yes	8	no DM	50 mg	0	sucrose	50	180	yes	yes
Lembcke 1991a (3)	(58)	cross-over	yes	8	no DM	100 mg	0	sucrose	50	180	yes	yes

Lembcke 1991a (4)	(58)	cross-over	yes	8	no DM	25 mg	0	starch	50	180	yes	yes
Lembcke 1991a (5)	(58)	cross-over	yes	8	no DM	50 mg	0	starch	50	180	yes	yes
Lembcke 1991a (6)	(58)	cross-over	yes	8	no DM	100 mg	0	starch	50	180	yes	yes
Masuda 2011 (1)	(59)	cross-over	yes	10	no DM	50 mg	0	mixed meal	121.5	180	yes	no
Masuda 2011 (2)	(59)	cross-over	yes	10	no DM	50 mg	2	mixed meal	121.5	180	yes	no
Renard 1991	(60)	cross-over	yes	16	no DM	100 mg	1	sucrose	75	120	yes	yes
Scott 1988 (1)	(61)	cross-over	yes	20	DM	50 mg	1	mixed meal	75	240	yes	yes
Scott 1988 (2)	(61)	cross-over	yes	20	DM	100 mg	1	mixed meal	75	240	yes	yes
Scott 1988 (3)	(61)	cross-over	yes	20	DM	150 mg	1	mixed meal	75	240	yes	yes
Scott 1988 (4)	(61)	cross-over	yes	20	DM	200 mg	1	mixed meal	75	240	yes	yes
Shibata 2016 (1)	(62)	Cross-over	Partial	13	DM	50 mg	0	Mixed meal	108	180	Yes	Yes
Shibata 2016 (2)	(62)	Cross-over	partial	13	DM	50 mg	1	Mixed meal	108	180	yes	Yes
Ueno 2015 (1)	(36)	cross-over	yes	11	DM	50 mg	0	mixed meal	71.4	180	yes	yes
Wing 1990	(63)	cross-over	yes	11	DM	50 mg	1	mixed meal	45	180	yes	no

Supplementary Table 1c: Characteristics of Study Comparisons for Voglibose

Author	ref	Study Design	Randomised	N	Health status	Dose	Timing (0-before; 1-with)	Meal test	Carbs (g)	Duration (min)	PPG	PPI
Aoki 2009 (1)	(64)	cross-over	yes	16	no DM	0.3 mg	0	mixed meal		180	yes	yes
Aoki 2009 (2)	(64)	cross-over	yes	16	no DM	0.3 mg	2	mixed meal		180	yes	yes
Goke 1995 (1)	(65)	parallel	yes	12	no DM	0.5 mg	1	mixed meal	111	180	yes	yes
Goke 1995 (2)	(65)	parallel	yes	12	no DM	1.0 mg	1	mixed meal	111	180	yes	yes
Goke 1995 (3)	(65)	parallel	yes	12	no DM	2.0 mg	1	mixed meal	111	180	yes	yes
Goke 1995 (4)	(65)	parallel	yes	12	no DM	5.0 mg	1	mixed meal	111	180	yes	yes
Hiki 2010 (2)	(50)	cross-over	yes	11	DM	0.3 mg	0	mixed meal	56.5	120	yes	yes

Kageyama 1997 (3)	(21)	cross-over	yes	20	no DM	0.2 mg	0	mixed meal	82.4	120	yes	yes
Kageyama 1997 (4)	(21)	cross-over	yes	20	no DM	0.3 mg	0	mixed meal	82.4	120	yes	yes
Kageyama 2000 (2)	(22)	cross-over	yes	12	no DM	0.3 mg	0	mixed meal	82.4	120	yes	yes

Supplementary Table 1d: Characteristics of Study Comparisons for Emiglitate

Author	ref	Study Design	Randomised	N minus dropout	Health status	Dose	Timing (0-before; 1-with)	Meal test	Carbs (g)	Duration (min)	PPG	PPI
Kennedy 1987 (3)	(55)	cross-over	yes	7	no DM	10 mg	1	mixed meal	101.3	180	yes	yes
Kennedy 1987 (4)	(55)	cross-over	yes	8	no DM	20 mg	1	mixed meal	101.3	180	yes	yes
Lembcke 1991b (1)	(66)	cross-over	yes	8	no DM	10 mg	0	sucrose	50	180	yes	yes
Lembcke 1991b (2)	(66)	cross-over	yes	8	no DM	20 mg	0	sucrose	50	180	yes	yes
Lembcke 1991b (3)	(66)	cross-over	yes	8	no DM	40 mg	0	sucrose	50	180	yes	yes
Lembcke 1991b (4)	(66)	cross-over	yes	8	no DM	10 mg	0	starch	50	180	yes	yes
Lembcke 1991b (5)	(66)	cross-over	yes	8	no DM	20 mg	0	starch	50	180	yes	yes
Lembcke 1991b (6)	(66)	cross-over	yes	8	no DM	40 mg	0	starch	50	180	yes	yes

Supplementary Table 2: Absolute and Relative Effects of Acarbose and Miglitol on PPG and PPI by Diabetes State by Fixed Effects Model

	**Mean PPG (mmol/l)[95%CI]**				**Mean PPI (pmol/l)[95%CI]**			
	**N[1]**	**No diabetes**	**N[2]**	**Diabetes**	**N[1]**	**No diabetes**	**N[2]**	**Diabetes**
**Acarbose**								
Mean difference, [95% CI]	30	-0.3 [-0.3, -0.2]	22	-1.8 [-1.9, -1.6]*	19	-50.6 [-56.1, -45.1]	15	-38.1 [-45.6, -30.5]*
Relative change, % [95% CI]	27	-46.1 [-48.8, -43.4]	19	-49.3 [-51.0, -47.6]	19	-67.4 [-68.6, -66.2]	15	-39.4 [-42.1, -36.6] *
**Miglitol**								
Mean difference, [95% CI]	16	-0.6 [-0.7, -0.5]	17	-1.6 [-1.8, -1.3]*	15	-17.7 [-22.4, -13.0]	13	-7.4 [-13.9, -0.9]*
Relative change, % [95% CI]	16	-57.8 [-61.8, -53.8]	17	-40.8 [-43.5, -38.1]*	15	-89.4 [-93.1, -85.7]	13	-23.7 [-29.2, -18.1]*

[1] N is the number of comparisons among non-diabetic individuals

[2] N is the number of comparisons among diabetic individuals

*P<0.05 for subgroup difference between no diabetes and diabetes by Chi^2

Supplementary data 1: Search String

Medline *via* Pubmed

"Glycoside Hydrolase Inhibitors"[Mesh] OR "Acarbose"[Mesh] OR "alpha-Glucosidases"[Mesh] OR "emiglitate" [Supplementary Concept] OR "miglitol" [Supplementary Concept] OR "voglibose" [Supplementary Concept] OR alpha-glucosidase[tiab] OR acarbose[tiab] OR miglitol[tiab] OR voglibose[tiab] OR emiglitate[tiab] OR glucobay[tiab] OR prandase[tiab] OR precose[tiab] OR glyset[tiab] OR "1 deoxynojirimycin"[tiab] OR afegostat[tiab] OR camiglibose[tiab] OR castanospermine[tiab] OR miglustat[tiab] OR neosalacinol[tiab] OR salaprinol[tiab] OR valiolamine[tiab]

AND

"Area Under Curve"[Mesh] OR "Postprandial Period"[Mesh] OR area under curve*[tiab] OR area under the curve[tiab] OR AUC[tiab] OR meal test*[tiab] OR postprandial[tiab] OR post-prandial[tiab] OR post meal[tiab] OR post-breakfast[tiab] OR post-lunch[tiab] OR daytime[tiab] OR continuous glucose monitoring[tiab] OR interstitial glucose[tiab] OR glucose profile[tiab] OR insulin profile[tiab]

AND

(intervention*[tiab] OR randomized controlled trial[pt] OR controlled clinical trial[pt] OR randomized controlled trials[mh] OR random allocation[mh] OR double-blind method[mh] OR single-blind method[mh] OR clinical trial[pt] OR clinical trials[mh] OR "clinical trial"[tw] OR ((singl*[tw] OR doubl*[tw] OR trebl*[tw] OR tripl*[tw]) AND (mask*[tw] OR blind*[tw])) OR "latin square"[tw] OR placebos[mh] OR placebo*[tw] OR random*[tw] OR research design[mh:noexp] OR comparative study[pt] OR evaluation studies[pt] OR cross-over studies[mh] OR control[tw] OR controll*[tw] OR prospectiv*[tw] OR volunteer*[tw])

AND

NOT (animals[mh] NOT humans[mh])

NOT (animal*[tiab] OR mice[tiab] OR mouse[tiab] OR rats[tiab] OR rat[tiab] OR dog[tiab] OR pig[tiab] OR dogs[tiab] OR animal*[tiab] OR pigs[tiab])

Embase

'glycosidase inhibitor'/exp OR 'acarbose'/exp OR 'alpha glucosidase'/exp OR 'alpha-glucosidase':ab,ti,kw OR acarbose:ab,ti,kw OR miglitol:ab,ti,kw OR voglibose:ab,ti,kw OR emiglitate:ab,ti,kw OR glucobay:ab,ti,kw OR prandase:ab,ti,kw OR precose:ab,ti,kw OR glyset:ab,ti,kw OR '1 deoxynojirimycin':ab,ti,kw OR afegostat:ab,ti,kw OR camiglibose:ab,ti,kw OR castanospermine:ab,ti,kw OR miglustat:ab,ti,kw OR neosalacinol:ab,ti,kw OR salaprinol:ab,ti,kw OR valiolamine:ab,ti,kw

AND

'area under the curve'/exp OR 'postprandial state'/exp OR ‘area under curve*’:ab,ti,kw OR ‘area under the curve*’:ab,ti,kw OR AUC:ab,ti,kw OR ‘meal test*’:ab,ti,kw OR postprandial:ab,ti,kw OR ‘post-prandial’:ab,ti,kw OR ‘post meal’:ab,ti,kw OR ‘post-breakfast’:ab,ti,kw OR ‘post-lunch’:ab,ti,kw OR daytime:ab,ti,kw OR ‘continuous glucose monitoring’:ab,ti,kw OR ‘interstitial glucose’:ab,ti,kw OR ‘glucose profile’:ab,ti,kw OR ‘insulin profile’:ab,ti,kw

AND

'clinical trial'/exp OR 'triple blind procedure'/exp OR 'double blind procedure'/exp OR 'single blind procedure'/exp OR 'randomization'/exp OR 'placebo'/exp OR 'methodology'/de OR 'comparative study'/de OR 'evaluation study'/de OR 'crossover procedure'/exp OR 'clinical trial':ab,ti,kw OR (singl*:ab,ti,kw OR doubl*:ab,ti,kw OR trebl*:ab,ti,kw OR tripl*:ab,ti,kw AND (mask*:ab,ti,kw OR blind*:ab,ti,kw)) OR 'latin square':ab,ti,kw OR placebo*:ab,ti,kw OR random*:ab,ti,kw OR control:ab,ti,kw OR controll*:ab,ti,kw OR prospectiv*:ab,ti,kw OR volunteer*:ab,ti,kw

AND

'human'/de AND ('article'/it OR 'article in press'/it OR 'conference paper'/it OR 'review'/it)

AND

NOT (mice:ab,ti OR mouse:ab,ti OR rats:ab,ti OR rat:ab,ti OR dog:ab,ti OR pig:ab,ti OR dogs:ab,ti OR animal*:ab,ti OR pigs:ab,ti)

Supplementary data 2: References for the 66 Publications Included in the Systematic Review

1. Arakawa ME, C.; Mita, T.; Fujitani, Y.; Shimizu, T.; Watada, H.; Kawamori, R.; Hirose, T. Miglitol suppresses the postprandial increase in interleukin 6 and enhances active glucagon-like peptide 1 secretion in viscerally obese subjects. Metabolism. 2008;57(9):1299-306.
2. Aziz TA. Effect of benfotiamine and silibininon postprandial hyperglycemia of healthy subjects challenged with sucrose load: Comparative study with acarbose. International Journal of Pharmacy and Pharmaceutical Sciences. 2013;5(4):530-3.
3. Caspary WF. Sucrose malabsorption in man after ingestion of alpha-glucosidehydrolase inhibitor. Lancet. 1978;1(8076):1231-3.
4. Ceriello AT, C.; Tonutti, L.; Giacomello, R.; Stel, L.; Motz, E.; Pirisi, M. Post-meal coagulation activation in diabetes mellitus: the effect of acarbose. Diabetologia. 1996;39(4):469-73.
5. Chen ZF, X.; Kuang, J.; Chen, J.; Chen, H.; Pei, J.; Yang, H. Single-dose acarbose decreased glucose-dependent insulinotropic peptide and glucagon levels in Chinese patients with newly diagnosed type 2 diabetes mellitus after a mixed meal. BMC Endocr Disord. 2016;16(1):55.
6. Debeljak J, Ferk P, Cokolic M, Zavratnik A, Tavc Benkovic E, Kreft S, et al. Randomised, double blind, cross-over, placebo and active controlled human pharmacodynamic study on the influence of silver fir wood extract (Belinal) on post-prandial glycemic response. Pharmazie. 2016;71(10):566-9.
7. DeLeon MJC, V.; Albert, S. G.; Mooradian, A. D. Glucagon-like peptide-1 response to acarbose in elderly type 2 diabetic subjects. Diabetes Res Clin Pract. 2002;56(2):101-6.
8. Dimitriadis GT, P.; Go, V.; Gerich, J. Effects of the disaccharidase inhibitor acarbose on meal and intravenous glucose tolerance in normal man. Metabolism. 1982;31(8):841-3.
9. Enc FYI, N.; Akin, L.; Turoglu, T.; Dede, F.; Haklar, G.; Tekesin, N.; Bekiroglu, N.; Yegen, B. C.; Rehfeld, J. F.; Holst, J. J.; Ulusoy, N. B. Inhibition of gastric emptying by acarbose is correlated with GLP-1 response and accompanied by CCK release. Am J Physiol Gastrointest Liver Physiol. 2001;281(3):G752-63.
10. Frank M. Effect of acarbose on the need for between-meal snacking in patients with type 1 diabetes: A placebo-controlled, double-blind, cross-over study. Diabetes, Nutrition and Metabolism - Clinical and Experimental. 1998;11(3):169-74.
11. Frati Munari AC. Lowering glycemic index of food by acarbose and Plantago psyllium mucilage. Arch Med Res. 1998;29(2):137-41.
12. Gentilcore DB, B.; Wishart, J. M.; Morris, H. A.; Horowitz, M.; Jones, K. L. Acarbose attenuates the hypotensive response to sucrose and slows gastric emptying in the elderly. Am J Med. 2005;118(11):1289.
13. Hermanns NB, A.; Haak, T. The addition of acarbose to insulin lispro reduces acute glycaemic responses in patients with type-2 diabetes. Exp Clin Endocrinol Diabetes. 2004;112(6):310-4.
14. Hillebrand IB, K.; Frank, G.; Fink, H.; Berchtold, P. The effects of the alpha-glucosidase inhibitor BAY g 5421 (Acarbose) on meal-stimulated elevations of circulating glucose, insulin, and triglyceride levels in man. Res Exp Med (Berl). 1979;175(1):81-6.
15. Hillebrand IB, K.; Frank, G.; Fink, H.; Berchtold, P. The effects of the alpha-glucosidase inhibitor BAY g 5421 (Acarbose) on postprandial blood glucose, serum insulin, and triglyceride levels: dose-time-response relationships in man. Res Exp Med (Berl). 1979;175(1):87-94.
16. Hopfner MD, B.; Spengler, M.; Folsch, U. R. Effect of acarbose and simultaneous antacid therapy on blood glucose. Arzneimittelforschung. 1997;47(10):1108-11.
17. Hucking KK, Z.; Pox, C.; Ritzel, R.; Holst, J. J.; Schmiegel, W.; Nauck, M. A. alpha-Glucosidase inhibition (acarbose) fails to enhance secretion of glucagon-like peptide 1 (7-36 amide) and to delay gastric emptying in Type 2 diabetic patients. Diabet Med. 2005;22(4):470-6.
18. Jenkins DJ, Taylor RH, Nineham R, Goff DV, Bloom SR, Sarson D, et al. Combined use of guar and acarbose in reduction of postprandial glycaemia. Lancet. 1979;2(8149):924-7.

19. Jenney A, Proietto J, O'Dea K, Nankervis A, Traianedes K, D'Embden H. Low-dose acarbose improves glycemic control in NIDDM patients without changes in insulin sensitivity. Diabetes Care. 1993;16(2):499-502.
20. Kado SM, T.; Aoki, A.; Nagase, T.; Katsura, Y.; Noritake, M.; Matsuoka, T.; Nagata, N. Effect of acarbose on postprandial lipid metabolism in type 2 diabetes mellitus. Diabetes Res Clin Pract. 1998;41(1):49-55.
21. Kageyama SN, N.; Sekino, H.; Nakano, S. Comparison of the effects of acarbose and voglibose in healthy subjects. Clin Ther. 1997;19(4):720-9.
22. Kageyama S. Comparison of the effects of acarbose and voglibose on plasma glucose, endogenous insulin sparing, and gastrointestinal adverse events in obese subjects: A randomized, placebo-controlled, double-blind, three-way crossover study. Current Therapeutic Research - Clinical and Experimental. 2000;61(9):630-45.
23. Koytchev R. Influence of acarbose on blood glucose and breath hydrogen after carbohydrate load with sucrose or starch. Arzneimittelforschung. 2009;59(11):557-63.
24. Kusunoki YK, T.; Myojin, M.; Miyakoshi, K.; Ikawa, T.; Matsuo, T.; Ochi, F.; Tokuda, M.; Murai, K.; Miuchi, M.; Hamaguchi, T.; Miyagawa, J.; Namba, M. Effect of additional administration of acarbose on blood glucose fluctuations and postprandial hyperglycemia in patients with type 2 diabetes mellitus under treatment with alogliptin. Endocr J. 2013;60(4):431-9.
25. Lee SC, J. Y.; Hong, K. S.; Yang, S. H.; Byun, S. Y.; Lim, H. S.; Shin, S. G.; Jang, I. J.; Yu, K. S. Pharmacodynamic comparison of two formulations of Acarbose 100-mg tablets. J Clin Pharm Ther. 2012;37(5):553-7.
26. Lettieri JTD, B. Effects of beano on the tolerability and pharmacodynamics of acarbose. Clin Ther. 1998;20(3):497-504.
27. O'Dea KT, J. Optimum effectiveness of intestinal alpha-glucosidase inhibitors: importance of uniform distribution through a meal. Am J Clin Nutr. 1985;41(3):511-6.
28. Ogawa ST, K.; Ito, S. Acarbose lowers serum triglyceride and postprandial chylomicron levels in type 2 diabetes. Diabetes Obes Metab. 2004;6(5):384-90.
29. Rabasa-Lhoret RB, Y.; Ducros, F.; Bourque, J.; Lavoie, C.; Massicotte, D.; Peronnet, F.; Chiasson, J. L. Use of an alpha-glucosidase inhibitor to maintain glucose homoeostasis during postprandial exercise in intensively treated Type 1 diabetic subjects. Diabet Med. 2001;18(9):739-44.
30. Rosak CH, E.; Walter, T.; Werner, J. The effect of combination treatment with acarbose and glibenclamide on postprandial glucose and insulin profiles: additive blood glucose lowering effect and decreased hypoglycaemia. Diabetes Nutr Metab. 2002;15(3):143-51.
31. Seifarth CB, J.; Holst, J. J.; Ritzel, R.; Schmiegel, W.; Nauck, M. A. Prolonged and enhanced secretion of glucagon-like peptide 1 (7-36 amide) after oral sucrose due to alpha-glucosidase inhibition (acarbose) in Type 2 diabetic patients. Diabet Med. 1998;15(6):485-91.
32. Shibao CG, A.; Diedrich, A.; Dossett, C.; Choi, L.; Farley, G.; Biaggioni, I. Acarbose, an alpha-glucosidase inhibitor, attenuates postprandial hypotension in autonomic failure. Hypertension. 2007;50(1):54-61.
33. Shimabukuro MH, N.; Chinen, I.; Yamakawa, K.; Takasu, N. Effects of a single administration of acarbose on postprandial glucose excursion and endothelial dysfunction in type 2 diabetic patients: a randomized crossover study. J Clin Endocrinol Metab. 2006;91(3):837-42.
34. Tamasawa AM, K.; Hariya, N.; Saito, M.; Ishida, H.; Doguchi, S.; Yanagiya, S.; Osonoi, T. Hydrogen gas production is associated with reduced interleukin-1beta mRNA in peripheral blood after a single dose of acarbose in Japanese type 2 diabetic patients. Eur J Pharmacol. 2015;762:96-101.
35. Taylor RH, Jenkins DJ, Barker HM, Fielden H, Goff DV, Misiewicz JJ, et al. Effect of acarbose on the 24-hour blood glucose profile and pattern of carbohydrate absorption. Diabetes Care. 1982;5(2):92-6.
36. Ueno HT, W.; Wang, H. W.; Yamashita, E.; Tsubouchi, C.; Nagamine, K.; Sakoda, H.; Nakazato, M. Effects of Miglitol, Acarbose, and Sitagliptin on Plasma Insulin and Gut Peptides in Type 2 Diabetes Mellitus: A Crossover Study. Diabetes Ther. 2015;6(2):187-96.

37. Uttenthal LOU, O. O.; Wood, S. M.; Ghiglione, M.; Ghatei, M. A.; Trayner, I. M.; Bloom, S. R. Long-term effects of intestinal alpha-glucosidase inhibition on postprandial glucose, pancreatic and gut hormone responses and fasting serum lipids in diabetics on sulphonylureas. Diabet Med. 1986;3(2):155-60.
38. Uttenthal LOU, O. O.; Ghiglione, M.; Bloom, S. R. Acute and short term effects of intestinal alpha-glucosidase inhibition on gut hormone responses in man. Dig Dis Sci. 1987;32(2):139-44.
39. Wachters-Hagedoorn REP, M. G.; Heimweg, J. A.; Heiner, A. M.; Elzinga, H.; Stellaard, F.; Vonk, R. J. Low-dose acarbose does not delay digestion of starch but reduces its bioavailability. Diabet Med. 2007;24(6):600-6.
40. Walton RJS, I. T.; Noy, G. A.; Alberti, K. G. Improved metabolic profiles in insulin-treated diabetic patients given an alpha-glucosidehydrolase inhibitor. Br Med J. 1979;1(6158):220-1.
41. Yuhong HW, F.; Yanfen, L.; Yu, L.; Ziqiang, L.; Liu, Y.; Shirong, L.; Jinxia, S.; Na, L.; Baohe, W.; Xiumei, G.; Deqin, Z. Comparison of the Effects of Acarbose and TZQ-F, a New Kind of Traditional Chinese Medicine to Treat Diabetes, Chinese Healthy Volunteers. Evid Based Complement Alternat Med. 2014;2014:308126.
42. Zulkarnain RS, A.; Setiabudy, R. Effect of a combination of Phaseolus vulgaris L. extract and acarbose on postprandial glucose level after cooked rice intake in healthy volunteers. Med J Indones. 2009;18:25-30.
43. Aoki KN, A.; Ito, S.; Nezu, U.; Iwasaki, T.; Takahashi, M.; Kimura, M.; Terauchi, Y. Administration of miglitol until 30 min after the start of a meal is effective in type 2 diabetic patients. Diabetes Res Clin Pract. 2007;78(1):30-3.
44. Aoki KK, H.; Terauchi, Y. Divided-dose administration of miglitol just before and 15 minutes after the start of a meal smoothes postprandial plasma glucose excursions and serum insulin responses in healthy men. Endocr J. 2007;54(6):1009-14.
45. Aoki KM, K.; Miyazaki, T.; Togashi, Y.; Terauchi, Y. Effects of miglitol, sitagliptin or their combination on plasma glucose, insulin and incretin levels in non-diabetic men. Endocr J. 2010;57(8):667-72.
46. Aoki KK, H.; Yoshimura, K.; Shibuya, M.; Masuda, K.; Terauchi, Y. Miglitol administered before breakfast increased plasma active glucagon-like peptide-1 (GLP-1) levels after lunch in patients with type 2 diabetes treated with sitagliptin. Acta Diabetol. 2012;49(3):225-30.
47. Aoki KK, H.; Masuda, K.; Kamiko, K.; Noguchi, Y.; Tajima, K.; Terauchi, Y. Effects of miglitol, vildagliptin, or their combination on serum insulin and peptide YY levels and plasma glucose, cholecystokinin, ghrelin, and obestatin levels. Endocr J. 2014;61(3):249-56.
48. Dimitriadis GH, E.; Alexopoulos, E.; Kordonouri, O.; Komesidou, V.; Ganotakis, M.; Raptis, S. Effects of alpha-glucosidase inhibition on meal glucose tolerance and timing of insulin administration in patients with type I diabetes mellitus. Diabetes Care. 1991;14(5):393-8.
49. Heinz GK, M.; Korn, A.; Waldhausl, W. Reduction of postprandial blood glucose by the alpha-glucosidase inhibitor Miglitol (BAY m 1099) in type II diabetes. Eur J Clin Pharmacol. 1989;37(1):33-6.
50. Hiki MS, K.; Kiyanagi, T.; Fukao, K.; Hirose, K.; Ohsaka, H.; Fukushima, Y.; Kume, A.; Matsumori, R.; Sumiyoshi, K.; Miyazaki, T.; Ohmura, H.; Kurata, T.; Miida, T.; Daida, H. Single administration of alpha-glucosidase inhibitors on endothelial function and incretin secretion in diabetic patients with coronary artery disease - Juntendo University trial: effects of miglitol on endothelial vascular reactivity in type 2 diabetic patients with coronary heart disease (J-MACH). Circ J. 2010;74(7):1471-8.
51. Joubert PHV, C. P.; Joubert, H. F.; Hillebrand, I. The effect of a 1-deoxynojirimycin derivative on post-prandial blood glucose and insulin levels in healthy black and white volunteers. Eur J Clin Pharmacol. 1985;28(6):705-8.
52. Joubert PHB, W. J.; Manyane, N. Effect of an alpha-glucosidase inhibitor (BAY m 1099) on post-prandial blood glucose and insulin in type II diabetics. Eur J Clin Pharmacol. 1986;30(2):253-5.
53. Kaku HT, Y.; Yamada, K. Anorexigenic effects of miglitol in concert with the alterations of gut hormone secretion and gastric emptying in healthy subjects. Horm Metab Res. 2012;44(4):312-8.

54. Katsilambros NP, P.; Toskas, A.; Protopapas, J.; Frangaki, D.; Marangos, M.; Siskoudis, P.; Anastasopoulou, K.; Xefteri, H.; Hillebrand, I. A double-blind study on the efficacy and tolerance of a new alpha-glucosidase inhibitor in type-2 diabetics. Arzneimittelforschung. 1986;36(7):1136-8.
55. Kennedy FPM, J. M.; Heiling, V.; Gerich, J. E. The effect of two new alpha-glucosidase inhibitors on metabolic responses to a mixed meal in normal volunteers. Clin Exp Pharmacol Physiol. 1987;14(8):633-40.
56. Kingma PJM, P. P.; Sels, J. P.; Nieuwenhuijzen Kruseman, A. C. alpha-Glucosidase inhibition by miglitol in NIDDM patients. Diabetes Care. 1992;15(4):478-83.
57. Lembcke BD, M.; Folsch, U. R.; Creutzfeldt, W. Postprandial glycemic control, hormonal effects and carbohydrate malabsorption during long-term administration of the alpha-glucosidase inhibitor miglitol. Digestion. 1990;47(1):47-55.
58. Lembcke BF, U. R.; Gatzemeier, W.; Ebert, R.; Siegel, E.; Creutzfeldt, W. Inhibition of glycemic and hormonal responses after repetitive sucrose and starch loads by different doses of the alpha-glucosidase inhibitor miglitol (BAY m 1099) in man. Pharmacology. 1991;43(6):318-28.
59. Masuda KA, K.; Terauchi, Y. Effects of miglitol taken just before or after breakfast on plasma glucose, serum insulin, glucagon and incretin levels after lunch in men with normal glucose tolerance, impaired fasting glucose or impaired glucose tolerance. J Diabetes Investig. 2011;2(6):435-40.
60. Renard EP-R, C.; Richard, J. L.; Jureidini, S.; Orsetti, A.; Mirouze, J. Effect of Miglitol (Bay m1099), a new alpha-glucosidase inhibitor, on glucose, insulin, C-peptide and GIP responses to an oral sucrose load in patients with post-prandial hypoglycaemic symptoms. Diabete Metab. 1991;17(3):355-62.
61. Scott ART, R. B. Alpha glucosidase inhibition in the treatment of non-insulin-dependent diabetes mellitus. Diabet Med. 1988;5(1):42-6.
62. Shibata E, Aoki K, Tajima K, Taguri M, Terauchi Y. Comparison of efficacy and safety of taking miglitol dissolved in water during a meal and taking a miglitol tablet just before a meal in patients with type 2 diabetes. Expert Opin Pharmacother. 2016;17(7):889-94.
63. Wing JK, W. J.; Berzin, M.; Diamond, T. H.; Griffiths, R. F.; Smit, A. M.; Osler, C. E. The acute effects of glucosidase inhibition on post-meal glucose increments in insulin-dependent diabetics. S Afr Med J. 1990;77(6):286-8.
64. Aoki KI, Y.; Saito, K.; Shirakawa, J.; Togashi, Y.; Satoh, K.; Muraoka, T.; Shinoda, K.; Masuda, K.; Kimura, M.; Terauchi, Y. Comparison of pre- versus post-meal administration of voglibose in men with or without impaired glucose tolerance. Diabetes Res Clin Pract. 2009;83(2):e31-2.
65. Goke BF, H.; Wieckhorst, G.; Theiss, U.; Stridde, E.; Littke, T.; Kleist, P.; Arnold, R.; Lucker, P. W. Voglibose (AO-128) is an efficient alpha-glucosidase inhibitor and mobilizes the endogenous GLP-1 reserve. Digestion. 1995;56(6):493-501.
66. Lembcke BF, U. R.; Gatzemeier, W.; Lucke, B.; Ebert, R.; Siegel, E.; Creutzfeldt, W. Inhibition of sucrose- and starch-induced glycaemic and hormonal responses by the alpha-glucosidase inhibitor emiglitate (BAY o 1248) in healthy volunteers. Eur J Clin Pharmacol. 1991;41(6):561-7.
